# Supplementary material for: Anticipated significant work limitation in primary care consulters with osteoarthritis: a prospective cohort study
Source: BMJ Open. 2014 Sep 4;4(9):e005221. doi: 10.1136/bmjopen-2014-005221 (PMC4158206; doi:10.1136/bmjopen-2014-005221)
Supplement: Author's manuscript [file bmjopen-2014-005221.draft_revisions.pdf]

Anticipated significant work limitation in primary care consulters with osteoarthritis: a prospective cohort study

Journal:	*BMJ Open*
Manuscript ID:	bmjopen-2014-005221
Article Type:	Research
Date Submitted by the Author:	07-Mar-2014
Complete List of Authors:	Wilkie, Ross; Keele University, Research Institute for Primary Care and Health Sciences<br>Phillipson, Christopher; University of Manchester, School of Social Sciences<br>Hay, Elaine; Keele University, Research Institute for Primary Care & Health Sciences<br>Pransky, Glenn; Liberty Mutual Research Institute,
**Primary Subject Heading**:	General practice / Family practice
Secondary Subject Heading:	Epidemiology, Rheumatology, Occupational and environmental medicine
Keywords:	PRIMARY CARE, EPIDEMIOLOGY, OCCUPATIONAL & INDUSTRIAL MEDICINE, Musculoskeletal disorders < ORTHOPAEDIC & TRAUMA SURGERY, RHEUMATOLOGY

SCHOLARONE™
Manuscripts

For peer review only

For peer review only - http://bmjopen.bmj.com/site/about/guidelines.xhtml

Anticipated significant work limitation in primary care consulters with osteoarthritis: a prospective cohort study

Ross Wilkie[1], Chris Phillipson[2], Elaine M Hay[1], Glenn Pransky[3]

[1]Arthritis Research UK Primary Care Centre, Primary Care Sciences, Keele University, Keele, Staffordshire, United Kingdom, ST5 5BG.
[2] School of Social Sciences, The University of Manchester, Manchester, United Kingdom, M13 9PL
[3] Center for Disability Research, Liberty Mutual Research Institute, 71 Frankland Rd., Hopkinton MA 01748. United States.

Address for reprint requests and for corresponding author
Dr. Ross Wilkie
Arthritis Research UK Primary Care Centre, Primary Care Sciences, Keele University, Keele, Staffordshire, United Kingdom, ST5 5BG.
Email: r.wilkie@keele.ac.uk
Tel: +44 1782 734845
Fax: +44 1782 583911

Short title: Expected work loss in osteoarthritis consulters

Key indexing terms: osteoarthritis, work disability, epidemiology, primary health care, joint pain

For peer review only

For peer review only - http://bmjopen.bmj.com/site/about/guidelines.xhtml

Abstract

Objective: To describe the prevalence of expected work limitations (EWL) prior to future retirement age in osteoarthritis consulters, and the associated health, socio-demographic and workplace factors.

Design: Population-based prospective cohort study.

Setting: General practices in Staffordshire, England.

Participants: 297 working adults aged 50 to 65, who had consulted primary care for osteoarthritis.

Outcome: EWL was defined using a single question, "Do you think joint pain will limit your ability to work before you reach 69 years old".

Results: 51 (17.2%) indicated that joint pain would not limit their ability to work until 69, 79 (26.6%) indicated EWL and 167 (56.2%) did not know if joint pain would limit work before 69. In bivariate analysis, physical function (Odds ratio 0.93; 95% confidence interval 0.91, 0.96), depression (4.51; 1.81, 11.3), cognitive complaint (3.84; 1.81, 8.18), current smoker (2.75; (1.02, 7.38), age (0.69; 0.58, 0.82), physically-demanding job (3.18; 1.50, 6.72), no opportunities to retrain (3.01; 1.29, 7.05) and work dissatisfaction (3.69; 1.43, 9.49) were associated with EWL. The final multivariate model, included physical function and age.

For peer review only - http://bmjopen.bmj.com/site/about/guidelines.xhtml

Conclusions: Only one in five osteoarthritis consulters expected that joint pain would not limit their work participation before 69 years old. Given the expectation for people to work until they're older, the results highlight the increasing need for clinicians to include work participation in their consultation and implement strategies to address work limitation. Targeting pain related functional limitation and effective communication with employers to manage workplace issues could reduce EWL.

Article summary

- Osteoarthritis is a common reason for primary care consultation and joint pain is strongly associated with ageing and disability. Loss of work participation is one form of disability that will become more important as adults work to older ages, due to rising state pension age, and have greater financial needs resulting from inadequate retirement resources.
- The aim of this study was to estimate the proportion of working age adults with osteoarthritis who predict that joint pain would limit their work or stop them working prior to a possible future pension age of 69 (i.e. EWL) and identify health, socio-demographic and workplace factors associated with EWL.

Key messages

- Only one in five consulters for osteoarthritis expected that joint pain would not limit their work participation before 69 years old.
- Low physical function, being a current smoker, depression, and several workplace factors - physically-demanding job, work dissatisfaction, and poor co-worker support – were associated with anticipated early work limitation.

Strengths and Limitations

For peer review only - http://bmjopen.bmj.com/site/about/guidelines.xhtml

- The sample is representative of primary care consulters with physician diagnosed osteoarthritis.
- The outcome is based on individual's expectations but this can be highly predictive of future work loss and drive consultation for health care.

For peer review only

Introduction

Osteoarthritis is the most common joint condition in adults and globally is the fastest increasing major heath condition [1]. It is a common reason for primary care consultation (one in twenty consultations in adults aged between 45 and 65 per year is for osteoarthritis [2]) and is recognised as one of the leading and rapidly growing causes of disability [3]. Its most disabling manifestation (joint pain) is strongly associated with ageing [4] and with the commonest forms of disability [5-9].

Work restriction is one form of disability that will become more important for those with osteoarthritis and joint pain because increases in state pension age in many developed countries means that most adults can expect a need to continue working at older ages than before [10]. Normal retirement age in North America and Europe has increased, and is expected to rise further to 69 and beyond [11]. However the extent to which participation in work will be limited by health-related problems, resulting in significant work limitation in terms of absenteeism and presenteeism (remaining in work but with limitation and reduced productivity) is unclear [12]. The increasing prevalence of chronic health conditions, especially osteoarthritis, in persons near to retirement age raises questions about viability of attempts to extend working life. Several studies of expectations of future work loss are

For peer review only - http://bmjopen.bmj.com/site/about/guidelines.xhtml

predictive of future work outcomes [13,14]. Identifying the prevalence and predictors of expected work limitations (EWL) in this group of patients, particularly those that are amenable to change, will inform management and possible preventative strategies for future work limitation. The aim of this study was to estimate the proportion of working age adults with osteoarthritis who predict that joint pain would limit their work or stop them working (i.e. EWL) prior to a possible future pension age of 69. In addition health, socio-demographic or workplace factors associated with EWL, especially those amenable to change, were explored, to identify potential targets to manage and prevent EWL [15-17].

Method

Study population

The North Staffordshire Osteoarthritis project (NorStOP) is a population-based prospective cohort study. The NorStOP sampling frame comprised all individuals aged 50 years and over who were registered to receive care from one of six general practices in North Staffordshire, England, United Kingdom (UK). In 2002, adults aged who gave their written consent for medical record review were followed up over 6 years for consultation to primary care. They were also mailed questionnaires at three and six years; reminders were sent at two and four weeks after the initial mailing. The North Staffordshire Local Research Ethics Committee approved this study.

Analyses for this paper included those who (i) consulted for osteoarthritis during the study period (starting 18 months before the baseline questionnaire was administered, and continuing through the time of the final follow-up questionnaire (i.e. from 2000 to 2008)), (ii) were of working age (less than 65 years old) and in employment at the six year follow-up and (iii) completed the item on expected work limitation prior to 69 years old (EWL) at six year follow-up. Over the study period there were 923 adults who had consulted for osteoarthritis

For peer review only - http://bmjopen.bmj.com/site/about/guidelines.xhtml

and were of working age at 6 year follow-up. Of this group 398 had retired before state retirement age, 13 were unemployed, 31 were homemakers, leaving 481 who were in employment and thus eligible for the study. Of these, 184 did not complete the item on future work limitation, leaving complete data for 297 participants (adjusted response 61.7%) (Figure 1). Compared to those subjects who had consulted for osteoarthritis but did not complete the item on future work limitation questionnaire (n=184) those included in the analysis (n=297) were more likely to be female (p=0.02) but no more likely to be older (p=0.19), have better physical (p=0.91) or mental health (p=0.21), have gone onto further education (p=0.52) or have an adequate income (p=0.052).

Identification of osteoarthritis

General practitioners in the study used the READ system to code all reasons for clinical encounters in primary care consultations [18]. The Read codes cross-map to ICD9/ICD-10 (for diseases). Morbidity data (i.e. symptoms and diseases) in this system are grouped under 19 main READ chapters. Data collected at the second hierarchical level or above was used to identify diagnostic groups, and these were aggregated starting 18 months before the baseline questionnaire was administered, and continuing through the time of the final follow-up questionnaire. Individuals were defined as having osteoarthritis if they had at least one consultation during this period primarily for osteoarthritis based on Read codes (N05 category) for primary care consultations [18].

Outcome measure

For peer review only - http://bmjopen.bmj.com/site/about/guidelines.xhtml

Expected work limitation (EWL) was defined using a single question at six-year follow-up, “do you think joint pain will limit your ability to work before you reach 69 years old” (will limit or stop me/ don’t know/ won’t limit).

Independent factors

Health factors were measured across the six year study period, socio-demographic factors at three year follow-up and workplace factors, were measured retrospectively at six year follow-up.

Health Factors

Physical function was measured at each time point using the physical functioning scale of the Medical Outcomes Study Short Form-36; score range: 0-100, higher scores indicating better function [19]. Items measured the limitation in the individual’s capacity to complete basic tasks such as lifting and walking. Scores at each time point were highly correlated (i.e. between baseline and three year follow-up r=0.71; between three and six year follow-up r=0.75). Physical function score at three year follow-up was used in this analysis. The extent of musculoskeletal pain was measured by responders shading painful areas (0-44) on a full body diagram (front and back views). These methods to determine the location and extent of pain are commonly used in population based studies of pain, and have been shown to be valid and reliable [20]. Using these pain drawings, participants were classified into one of three groups (none, some and widespread). The widespread group were those participants that satisfied the criteria for widespread pain included in the American College of Rheumatology 1990 criteria for fibromyalgia [21] at baseline, three or six years. These criteria require pain to be present above and below the waist, in the right and left hand sides of the body and in the axial skeleton; remaining participants who reported pain at any time point that did not satisfy

For peer review only - http://bmjopen.bmj.com/site/about/guidelines.xhtml

the criteria for widespread were classified as having “some pain” and those participants who did not report pain at all were classified as “no pain”.

Comorbidity was identified using Read diagnostic codes from primary care consultations. Multimorbidity was defined as 4 or more comorbidities (different major diagnostic groups) in the 2 years prior to baseline, between baseline and 3 year follow-up or between three and 6 year follow-up [22]. Anxiety and depression during the previous week were measured using the Hospital Anxiety and Depression Scale (HADS) - raw scores categorised individuals as non-cases (0-7) or possible/probable cases (8-21); depression was defined as possible or probable case at any of the three time points [23]. Self-reported height and weight was categorised into standard BMI groups (i) normal weight (BMI 20-24.9kgm^{-2}), (ii) underweight (BMI <20kgm^{-2}), (iii) overweight (BMI 25 – 29.9 kgm^{-2}) and (iv) obese (BMI ≥30kgm^{-2}). Cognitive complaint was measured using the Alertness Behaviour Subscale of the Sickness Impact Profile [24]. This scale has 10 items that ask about alertness and ability to concentrate. Each item was scored as 0 (no cognitive complaint) or 1 (cognitive complaint) with raw additive scores categorised to indicate “no cognitive complaint” (score of 0) and “cognitive complaint” (score >0); individuals were identified as having cognitive complaint if they had a score >0 at any of the three time points. Perceived control of health was measured using a single item at baseline (There is a lot I can do to control my health yes/no). Participants were also asked to report their smoking status at baseline (current, previous, never).

Socio-demographic Factors

Demographic and socio-economic details included age, gender, educational attainment (those who finished their education on leaving school; those who went onto further education such as college or university), occupational class (managerial or professional, intermediate (e.g.

For peer review only - http://bmjopen.bmj.com/site/about/guidelines.xhtml

For peer review only

paramedics, technicians), routine), adequacy of income (Thinking about the cost of living as it affects you, which of these descriptions best describes your situation; adequate/inadequate) and living status (live alone/live with others).

Workplace Factors

Single items on the 6-year follow-up questionnaire measured workplace characteristics. Items included work type (Which of the following statements best describes the work that you do in your current job; sedentary occupation/standing occupation/physical work/heavy manual work), work status (current employment status: full-time/part-time/temporarily off work (e.g. due to sickness), physically demanding employment (Thinking over the last 30 days: Is your work physically demanding; not physically demanding/physically demanding), flexible working (My hours of work are flexible; flexible/not flexible), co-worker support (My work colleagues are supportive: good/low), work satisfaction (How satisfied are you with your current job satisfaction/dissatisfied), opportunities to retrain (There are opportunities to retrain and develop my skills; yes/no) and able to use aids and appliances and adapt the workplace (I can use aids and appliances to help me do my job or adapt my work; yes/no).

Statistical analysis

First, the distribution of health, socio-demographic and workplace factors was examined by prediction of the ability to work until 69 with differences tested for significance using Chi-square or Kruskall Wallis tests where appropriate. Regression analyses then focused on identifying factors associated with significant future work loss (will be limited in ability to work, or will be unable to work until age 69 because of joint pain (i.e. EWL)); compared to no expected work loss. Bivariate and multivariate logistic regression models were constructed to examine the relationship between each health, socio-demographic and workplace factor and EWL. Following bivariate analysis, multivariate models included all of

For peer review only - http://bmjopen.bmj.com/site/about/guidelines.xhtml

the factors within each category (i.e. all health factors in one model, all socio-demographic in the second model and all of the workplace factors in the third model for each outcome). Factors independently associated with EWL within each category were then included in a final multivariate model. To evaluate model fit, concordance indexes (C-statistic) were calculated for each model. A C-statistic of 0.50 indicates the predictive ability of a model to be no better than chance, 0.7 indicates reasonable, 0.8 indicates high and 1.0 indicates perfect predictive ability [25].

Stata version 11 was used for all analyses. The results of the analyses are presented as odds ratios (OR) with 95% confidence intervals (CI). For the regression analyses the "won't be limited" group was classified as the referent category.

Results

Of the 297 consulters for osteoarthritis included in the analysis, 51 (17.2%) indicated that joint pain would not limit their ability to work until 69, 79 (26.6%) indicated that joint pain would limit or stop them working before 69 (EWL) and 167 (56.2%) indicated that they did not know if they would have EWL before 69. Those who indicated EWL (median age 53) were younger than those who did not know (median age 54) or did not predict EWL (median age 57; p=0.0001) (Table 1). Women were more likely to indicate that they did not know or would have EWL (p=0.01). There was no difference among the three groups for educational attainment (p=0.44) or occupational classification (p=0.10). Notably all responders with low co-worker support predicted developing EWL before age 69.

Table 2 shows the results of the regression analysis comparing anticipated EWL to no EWL. In the bivariate analysis, health, socio-economic and workplace factors were associated with

For peer review only - http://bmjopen.bmj.com/site/about/guidelines.xhtml

For peer review only

EWL before age 69. Of the health factors, depression (4.51; 1.81, 11.3), cognitive complaint (3.84; 1.81, 8.18) and being a current smoker (2.72; (1.02, 7.38) were associated with EWL onset prior to 69. Increasing physical function (0.93; 0.91, 0.96) was protective against EWL onset prior to 69. In the multivariate analysis of health factors, current smoker (6.85; 1.48, 31.7) and increasing physical function (0.94; 0.90, 0.97) were associated with EWL onset before 69. Of the socio-economic factors, only age (0.69; 0.58, 0.82) was associated with EWL. Of the workplace factors, a physical demanding job (3.18; 1.50, 6.72), no opportunities to retrain (3.01; 1.29, 7.05) and work dissatisfaction (3.69; 1.43, 9.49) were associated with EWL. In the multivariate analysis of workplace factors, a physically demanding job and work satisfaction were independently associated with EWL. In the final multivariate model, combining significant factors from the health, socio-economic and workplace factors, the association with work dissatisfaction attenuated to insignificance (2.08; 0.66, 6.51). Physical function (0.95; 0.92, 0.97) and age (0.74; 0.60, 0.91) remained independently associated with EWL. The model fit of the final model was 0.8418.

Discussion

Principal findings

This study is the first to examine the factors associated with an expectation of having significant future work limitation (EWL) for employed patients who consult primary care physicians for osteoarthritis. Physical function, being a current smoker, depression, and several workplace factors - physically-demanding job, work dissatisfaction, and poor co-worker support – were associated with anticipated EWL. Only one in five osteoarthritis consulters (17.2%) indicated that joint pain would not limit their ability to work until age 69. Given the high and increasing prevalence of osteoarthritis, the number of consultations for

For peer review only - http://bmjopen.bmj.com/site/about/guidelines.xhtml

this disorder, and the anticipated extension of working lives due to changes in retirement policy, these results raise significant concerns, while suggesting potential areas for intervention.

The proportion of osteoarthritis cases who expected work limitation was higher compared to previous studies. Using a national US sample of all workers, Theis and Murphy found that only a third of those with physician-diagnosed arthritis reported work limitations [15]. Unlike the current study, their sample was not restricted to employed persons, and did not ask about expected ability to work in the future. Studies of rheumatoid arthritis patients have found that premature work loss in persons in a similar age group is common, affecting up to 90% of persons with this diagnosis, and leads to significant economic and social consequences [26]. Our finding of a similar high rate of expected work loss in persons with OA suggests that the societal impact in this much larger group will be significant as well.

Physical function was highly protective against EWL, similar to findings observed in other studies of current work limitations in persons with any type of arthritis [15], musculoskeletal disorders in general [27] or those with a specific arthritis diagnosis [28,29]. This indicates an important mismatch between individual capabilities and work demands, that is more important than pain by itself. High work physical demands have long been recognized as an important risk factor for subsequent work disability in rheumatoid arthritis [27]. Some have suggested that control over work demands might be more important than the absolute level of work demands, but these studies did not evaluate the level of job physical demands [30]. Work dissatisfaction was also significant, although this problem was reported in relatively few respondents. There may be several dimensions of work dissatisfaction – not only being dissatisfied at work, but also a preference to be at home instead [30]. Similarly, lack of co-

For peer review only - http://bmjopen.bmj.com/site/about/guidelines.xhtml

worker support was a problem in only a relatively small number of persons, but all who reported low co-worker support expected to have EWL. All of those who reported low co-worker support also indicated at least one significant health problem (i.e. pain interference, multimorbidity, depression or anxiety). The importance of co-worker support in those returning to work after injury or illness, across a range of conditions, has recently been recognized [31], and low co-worker support has been associated with greater job strain and work loss in workers with arthritis [32,33]. This may not be about co-worker support but be a description of work, such as people working on their own, or in job types in which cannot involve others. If this changes in the future the implications of this will increase.

We did not see the effects of low education (e.g. [15]) or comorbidities reported by others. Education may not have a large impact compared to actual job physical demands. Flexible work was not a factor, although it has been identified in other studies as a significant predictor of staying at work with RA [33]. This difference may be due to the less specific nature of the question in this study, compared with other investigations.

The majority of participants (52.6%) could not predict whether pain would limit or stop their ability to work until the new retirement age. In additional analysis, a forward stepwise logistic regression model of health, socio-demographic and workplace factors was constructed to identify which factors were associated with being unable to predict whether pain would limit the ability to work.. Lower age, being in part time work and being unable to use aids and appliances were the factors significantly associated with being unable to predict. Their responses may be due to some uncertainty about the future progression of their condition, or about whether or not employment until age 69 would be required.

For peer review only - http://bmjopen.bmj.com/site/about/guidelines.xhtml

For peer review only

Notably the greater likelihood for women to indicate "don't know" was explained by these factors. The role of aids and appliances again indicates the importance of the workplace to allow individuals to self-manage their pain to optimise performance.

Strengths and weaknesses

The strength of this study's longitudinal design enables prospective identification of factors associated with EWL in a clinically relevant primary care population. The sample is representative of primary care consulters with physician diagnosed osteoarthritis, relevant to primary care practices. Other studies have been limited to patients from rheumatology practices or rehabilitation clinics, a less representative sample of osteoarthritis patients.

There are limitations to this study. A range of factors have been identified as potentially linked to future employment loss in osteoarthritis, and some were not included in the information collected for this study, such as extent of joint involvement, success of current coping strategies, opportunities for part-time work or retirement, importance of work role, social support outside of the workplace, and extent of workplace accommodations specific to their osteoarthritic condition [16]. However, clinical measures may not be as important in predicting work disability, compared to measure of function, psychosocial and workplace factors [34]. The C-statistics for the health (0.8641), socio-demographic (0.7636), workplace (0.7785) and final (0.8418) models indicate a reasonable or high ability to predict EWL. The factors included here are those thought to be most important in maintaining employment in chronic musculoskeletal conditions. Data on most variables was by self-report, but validated instruments were used to measure anxiety, depression and pain interference. The outcome is based on individual's expectations but this can be highly predictive of future work loss and drive consultation for health care [15-17]. We did not have radiographic or detailed

For peer review only - http://bmjopen.bmj.com/site/about/guidelines.xhtml

information on the extent of OA, but the intention of the study was to describe a typical, heterogeneous group of patients with OA as seen in primary care practice. Measurement of predictors at three time points may not reflect changes in these factors during follow-up. Workplace factors were measured only at six year follow-up, and may be out of sequence with health factors, but give a sense of current workplace status at the same time that the question on anticipated EWL was asked. As with any cohort study, non-completion of the items may affect estimates, however based on comparisons between those included in the analysis and those who did not complete the EWL item, such effects are likely to be small. The area covered by the study is more deprived on health, education, and employment, but with fewer barriers to housing and services, than England as a whole, but again the potential effect of this on estimates will be small.

Clinical implications

With current disease prevalence and economic trends, and increasing prevalence of osteoarthritis, clinicians will undoubtedly have more consultations with persons concerned about their ability to stay at work [35]. Studies in rheumatoid arthritis suggest that a proactive approach – identification and intervention before work loss has occurred – can be effective in preventing subsequent work loss [36]. In this study, the health and workplace problems were present well in advance of when EWL was measured. For example, the high correlation between physical function scores at baseline and three years (0.71) indicates that low physical function was experienced six years before EWL was measured. This indicates that there is a lot of time to intervene with available strategies [37]. Certain types of coping strategies may be more effective than others in maintaining employment in persons with arthritis, especially anticipating challenges in the workplace and formulating

For peer review only - http://bmjopen.bmj.com/site/about/guidelines.xhtml

strategies to deal with them in advance [38]. Given the importance of osteoarthritis-related physical limitations at work as a predictor of EWL, a positive screening question for these limitations should lead to a more in-depth discussion about accommodations and employment options, and perhaps a referral to a vocational expert. The negative association with inadequate co-worker support suggests that employer engagement in creating a supportive work environment will also be a major factor in work retention for these patients [33]. Addressing the needs of employed osteoarthritis patients within the context of a broader psychosocial model of disease ensures a broader and more relevant perspective on the causes and prevention opportunities for subsequent work loss [17]. There are still many unanswered questions about the optimal nature, timing and duration of interventions designed to maintain employment, and how to engage treating clinicians as positive contributors; thus further studies will be needed in order to understand how best to address these problems [39, 40].

Conclusion

This observational study suggests only one in five consulters to primary care with osteoarthritis do not expect their joint pain to limit their work participation prior to future pension age. Given the expectation for people to work until they are older with osteoarthritis, the results highlight the increasing need for clinicians to include work participation in their consultation and implement strategies to prevent work limitation. Targeting pain related functional limitation and effective communication with employers to manage workplace issues could reduce the expectation of future work limitation.

ACKNOWLEDGEMENTS

For peer review only

For peer review only - http://bmjopen.bmj.com/site/about/guidelines.xhtml

We would like to thank the administrative and health informatics staff at Keele University's Arthritis Research UK Primary Care Centre and the doctors and staff of the participating general practices.

FINANCIAL DISCLOSURE: This study is supported financially by the Medical Research Council, UK (grant code: G9900220) and by the North Staffordshire Primary Care R&D Consortium.

DATA SHARING: The Arthritis Research UK Primary Care Research Centre has established data sharing arrangements to support joint publications and other research collaborations. Applications for access to anonymized data from our research databases are reviewed by the Centre's Data Custodian and Academic Proposal (DCAP) Committee and a decision regarding access to the data is made subject to the NRES ethical approval first provided for the study and to new analysis being proposed. Further information on our data sharing procedures can be found on the Centre's website (http://www.keele.ac.uk/pchs/publications/datasharingresources/) or by emailing the Centre's data manager (data-sharing-pcs@cphc.keele.ac.uk).

For peer review only

For peer review only - http://bmjopen.bmj.com/site/about/guidelines.xhtml

Funding: The NorStOP study was funded by a programme Grant from the MRC UK (grant code G9900220)

Contributorship Statement: RW is the principal investigator of the project, responsible for the conception and design of the study. He was involved in the data analysis and led the drafting and final editing of the paper. CP and EM are guarantors and advised on the study design. GP is co-investigator of the project. He led on the data analysis and contributed to the drafting and final editing of the manuscript. All authors contributed to the final version of the paper.

Conflicts of interest: The authors declare no conflicts of interest

Data Sharing Statement: The Arthritis Research UK Primary Care Research Centre has established data sharing arrangements to support joint publications and other research collaborations. Applications for access to anonymized data from our research databases are reviewed by the Centre's Data Custodian and Academic Proposal (DCAP) Committee and a decision regarding access to the data is made subject to the NRES ethical approval first provided for the study and to new analysis being proposed. Further information on our data sharing procedures can be found on the Centre's website (http://www.keele.ac.uk/pchs/publications/datasharingresources/) or by emailing the Centre's data manager (data-sharing-pcs@cphc.keele.ac.uk).\

For peer review only

For peer review only - http://bmjopen.bmj.com/site/about/guidelines.xhtml

References

1. Murray CJ, Vos T, Lozano R, Naghavi M, Flaxman AD, Michaud C et al. Disability-adjusted life years (DALYs) for 291 diseases and injuries in 21 regions, 1990-2010: a systematic analysis for the Global Burden of Disease Study 2010. Lancet 2012;380(9859):2197-223.

2. Jordan K, Clarke AM, Symmons DP, Fleming D, Porcheret M, Kadam UT, Croft P. Measuring disease prevalence: a comparison of musculoskeletal disease using four general practice consultation databases. Br J Gen Pract 2007;57:7-14.

3. Arden N, Nevitt MC. Osteoarthritis: epidemiology. Best Pract Res Clin Rheumatol 2006;20:3-25.

4. Doherty M. Risk factors for progression of knee osteoarthritis. Lancet 2001;358:775-6.

5. Jordan JM, Luta G, Renner JB, Linder GF, Dragomir A, Hochberg MC, Fryer JG. Self-reported functional status in osteoarthritis of the knee in a rural southern community: the role of sociodemographic factors, obesity, and knee pain. Arthritis Care Res 1996;9:273-8.

6. Jinks C, Jordan K, Croft P. Osteoarthritis as a public health problem: the impact of developing knee pain on physical function in adults living in the community: (KNEST 3). Rheumatology 2007;46(5):877-81.

7. Felson DT, Lawrence RC, Dieppe PA, Hirsch R, Helmick CG, Jordan JM, Kington RS, Lane NE, Nevitt MC, Zhang Y, Sowers M, McAlindon T, Spector TD, Poole AR, Yanovski SZ, Ateshian G, Sharma L, Buckwalter JA, Brandt KD, Fries JF. Osteoarthritis: new insights. Part 1: the disease and its risk factors. Ann Intern Med 2000;133:635-46.

8. Wilkie R, Peat G, Thomas E, Croft PR. Factors associated with restricted mobility outside the home in community-dwelling adults aged 50 years and over with knee pain – an

For peer review only - http://bmjopen.bmj.com/site/about/guidelines.xhtml

example of use of the International Classification of Functioning to investigate participation restriction. Arthritis Rheum 2007;57(8):1381-9.

9. Odding E, Valkenburg HA, Stam HJ, Hofman A. Determinants of locomotor disability in people aged 55 years and over: The Rotterdam study. Eur J Epidemiol 2002;17:1033-41.

10. Helman R, Copeland C, VanDerhei J. The 2012 Retirement Confidence Survey: job insecurity, debt weigh on retirement confidence, savings. EBRI Issue Brief 2012;369:5-32,1.

11. Organisation for Economic Co-operation and Development. OECD Pension Outlook 2012. http://www.oecd.org/pensions/private-pensions/50560110.pdf (accessed August 2013).

12. Bieleman HJ, Bierma-Zeinstra SM, Oosterveld FG, Reneman MF, Verhagen AP, Groothoff JW. The effect of osteoarthritis of the hip or knee on work participation. J Rheumatol 2011;38(9):1835-43.

13. Fadyl J, McPherson K. Return to work after injury: a review of evidence regarding expectations and injury perceptions, and their influence on outcome. J Occup Rehabil. 2008;18(4):362-74.

14. Reme SE, Hagen EM, Eriksen HR. Expectations, perceptions, and physiotherapy predict prolonged sick leave in subacute low back pain. BMC Musculoskelet Disord 2009;10:139.

15. Theis KA, Murphy L, Hootman JM, Helmick CG, Yelin E. Prevalence and correlates of arthritis-attributable work limitation in the US population among persons ages 18-64: 2002 National Health Interview Survey Data. Arthritis Rheum 2007;57(3):355-63.

16. Hoving JL, van Zwieten MC, van der Meer M, Sluiter JK, Frings-Dresen MH. Work participation and arthritis: a systematic overview of challenges, adaptations and opportunities for interventions. Rheumatology 2013;52(7):1254-64

For peer review only

17. Wilkie R, Pransky G. Improving work participation for adults with musculoskeletal conditions. Best Pract Res Clin Rheumatol 2012;26(5):733-42.

18. NHS Information. The clinical terms version 3 (The READ Codes). Birmingham: NHS Information Authority, 2000.

19. Ware J Jr, Kosinski M, Keller SD. A 12-item Short-Form Health Survey: construction of scales and preliminary tests of reliability and validity. Med Care 1996;34:220-233.

20. Lacey RJ, Lewis M, Jordan K, Jinks C, Sim J. Interrater reliability of scoring of pain drawings in a self-report health survey. *Spine* 2005;30(16):E455-8.

21. Wolfe F, Smythe HA, Yunus MB, Bennett RM, Bombardier C, Goldenberg DL, et al. The American College of Rheumatology 1990 Criteria for the Classification of Fibromyalgia. Report of the Multicenter Criteria Committee. *Arthritis Rheum* 1990;33(2):160-72.

22. Kadam UT, Croft PR; North Staffordshire GP Consortium Group. Clinical multimorbidity and physical function in older adults: a record and health status linkage study in general practice. Fam Pract 2007;24:412-9.

23. Zigmond AS, Snaith RP. The hospital anxiety and depression scale. Acta Psychiatr Scand 1983;67:361-70.

24. Bergner M, Bobbitt RA, Carter WB, Gilson BS. The Sickness Impact Profile: development and final revision of a health status measure. *Med Care* 1981;19(8):787-805.

25. Hosmer DW, Lemeshow S. Applied Logistic Regression (2nd Edition). New York, NY: John Wiley & Sons; 2000.

26. Allaire S, Wolfe F, Niu J, Lavalley M, Michaud K. Work disability and its economic effect on 55-64-year-old adults with rheumatoid arthritis. Arthritis Rheum 2005;53(4):603-8.

For peer review only

For peer review only - http://bmjopen.bmj.com/site/about/guidelines.xhtml

27. Yelin EH, Henke CJ, Epstein WV: Work disability among Demons with musculoskeletal conditions. Arthritis Rheum 29: 1322-1333, 1986

28. Allaire SH, Anderson JJ, Meenan RF. Reducing work disability associated with rheumatoid arthritis: identification of additional risk factors and persons likely to benefit from intervention. Arthritis Care Res 1996;9(5):349-57.

29. Lacaille D, Sheps S, Spinelli JJ, Chalmers A, Esdaile JM. Identification of modifiable work-related factors that influence the risk of work disability in rheumatoid arthritis. Arthritis Rheum 2004;51:843–52.

30. Reisine S, McQuillan J, Fifield J. Predictors of work disability in rheumatoid arthritis patients. A five-year followup. Arthritis Rheum 1995;38(11):1630-7.

31. Dunstan DA, Maceachen E. A Theoretical Model of Co-worker Responses to Work Reintegration Processes. J Occup Rehabil. 2013 Aug 3.

32 Gignac MA, Sutton D, Badley EM. Arthritis symptoms, the work environment, and the future: measuring perceived job strain among employed persons with arthritis. Arthritis Rheum. 2007;57(5):738-47.

33. Lacaille D, White MA, Backman CL, Gignac MA. Problems faced at work due to inflammatory arthritis: new insights gained from understanding patients' perspective. Arthritis Rheum 2007;57(7):1269-79

34. Sokka T, Pincus T. Markers for work disability in rheumatoid arthritis. The Journal of Rheumatology 2001;28(7):1718–22.

35. Helmick CG, Felson DT, Lawrence RC, Gabriel S, Hirsch R, Kwoh CK, et al. Estimates of the prevalence of arthritis and other rheumatic conditions in the United States. Arthritis and Rheumatism 2008;58(1):15–25

For peer review only - http://bmjopen.bmj.com/site/about/guidelines.xhtml

36. Allaire SH, Li W, LaValley MP. Reduction of job loss in persons with rheumatic diseases receiving vocational rehabilitation: a randomized controlled trial. Arthritis Rheum 2003;48(11):3212-8.

37. Franche RL, Cullen K, Clarke J et al. Workplace-based return-to-work interventions: a systematic review of the quantitative literature. J Occup Rehabil 2005;15:607–631.

38. Gignac MA. Arthritis and employment: an examination of behavioral coping efforts to manage workplace activity limitations. Arthritis Rheum 2005;53(3):328-36.

39. Gignac MA, Jetha A, Bowring J, Beaton DE, Badley EM. Management of work disability in rheumatic conditions: a review of non-pharmacological interventions. Best Pract Res Clin Rheumatol. 2012;26(3):369-86.

40. Coole C, Watson PJ, Drummond A. Staying at work with back pain: patients' experiences of work-related help received from GPs and other clinicians. A qualitative study. BMC Musculoskelet Disord 2010;11:190.

Table 1. Subject characteristics overall and by prediction status (n=297)

	All (n=297)	Will work to 69 without limitation (n=51)	Don't know if joint pain will limit (n =167)	Joint pain will limited or stop me working until 69 (n=79)
Age				
Median (Standard error)	54 (0.25)	57 (0.35)	54 (0.18)	53 (0.22)
Gender				
Male	134 (45.1)	32 (60.0)	64 (38.3)	38 (48.1)
Female	163 (54.9)	19 (40.0)	103 (61.7)	41 (51.9)
Educational attainment				
Further	63 (21.5)	11 (21.6)	39 (23.8)	13 (16.7)
School only	230 (78.5)	40 (78.4)	125 (76.2)	65 (83.3)
Live alone				
No	37 (12.9)	6 (12.2)	14 (8.6)	17 (22.7)
Yes	250 (87.1)	43 (87.8)	149 (91.4)	58 (77.3)
Occupational classification				
Managerial/Professional	73 (24.7)	17 (33.3)	40 (24.1)	16 (20.3)
Intermediate	57 (19.3)	10 (19.6)	28 (16.9)	19 (24.1)
Routine	166 (56.1)	24 (47.1)	98 (59.0)	44 (55.7)
Adequacy of income				
Adequate	129 (43.4)	22 (43.1)	85 (50.9)	22 (27.9)
Inadequate	168 (56.6)	29 (56.9)	82 (49.1)	57 (72.2)
Physical Function				
Median (Standard error)	85 (10.5)	90 (7.91)	85 (9.27)	65 (12.2)
Pain status				
None	15 (5.1)	2 (3.9)	12 (7.2)	1 (1.3)
Some	143 (48.2)	30 (58.8)	88 (52.7)	25 (31.7)
Widespread	139 (46.8)	19 (37.3)	67 (40.1)	53 (67.1)
Comorbidity				

For peer review only - http://bmjopen.bmj.com/site/about/guidelines.xhtml

Low comorbidity (0-3)	122 (41.1)	20 (39.2)	78 (46.7)	24 (30.4)
Multimorbidity (4 or more)	175 (58.9)	31 (60.8)	89 (53.3)	55 (69.6)
Depression				
Non-case (0-7)	228 (76.8)	44 (86.3)	138 (82.6)	46 (58.2)
Possible/probable case (8-21)	69 (23.2)	7 (13.7)	29 (17.4)	33 (41)
Anxiety				
Non-case (0-7)	121 (40.7)	27 (52.9)	65 (38.9)	29 (36.7)
Possible/probable case (8-21)	176 (59.3)	24 (47.1)	102 (61.1)	50 (63.3)
Body mass Index				
Normal (20-24.9 kg m^2)	68 (24.7)	13 (27.7)	42 (26.4)	13 (18.8)
Underweight (<20)	9 (3.3)	1 (2.1)	6 (3.8)	2 (2.9)
Overweight (25-29.9 kg m^2)	102 (37.1)	21 (44.7)	52 (32.7)	29 (42.0)
Obese (>30 kg m^2)	96 (34.9)	12 (25.5)	59 (37.1)	25 (36.2)
Cognitive complaint				
No cognitive complaint	122 (41.8)	28 (54.9)	75 (44.9)	19 (24.1)
Cognitive complaint	175 (58.9)	23 (45.1)	92 (55.1)	60 (76.0)
Smoking				
Never	119 (40.2)	22 (44.0)	73 (43.7)	24 (30.4)
Previous	119 (40.2)	20 (40.0)	68 (40.7)	31 (39.2)
Current	58 (19.6)	8 (16.0)	26 (15.6)	24 (30.4)
Control				
Can control health	277 (93.9)	48 (94.1)	157 (95.2)	72 (91.1)
Can't control health	18 (6.1)	3 (5.9)	8 (4.9)	7 (8.9)
Work satisfaction				
Satisfied	198 (74.2)	43 (86.0)	115 (75.2)	40 (62.5)
Dissatisfied	69 (25.8)	7 (14.0)	38 (24.8)	24 (37.5)
Work type*				
Sedentary	88 (34.1)	18 (36.7)	53 (36.1)	17 (27.4)
Standing	71 (27.5)	9 (23.1)	41 (27.9)	21 (33.9)
Physical	81 (31.4)	20 (38.9)	45 (30.6)	16 (25.8)
Heavy manual	18 (7.0)	2 (4.6)	8 (5.4)	8 (12.9)
Physically demands of job				
Not physically demanding	171 (57.6)	36 (70.6)	101 (60.5)	34 (43.0)
Physically demanding	126 (42.4)	15 (29.4)	66 (39.5)	45 (57.0)
Flexible working				
Flexible	82 (27.6)	19 (37.3)	39 (23.4)	24 (30.4)
Not flexible	215 (72.4)	32 (62.8)	128 (76.7)	55 (69.6)
Use of aids and appliances				
Yes	151 (50.8)	32 (62.8)	79 (47.3)	40 (50.6)
No	146 (49.2)	19 (3.7.3)	88 (52.7)	39 (49.4)
Opportunities to retrain				
Yes	205 (69.0)	42 (82.4)	115 (68.9)	48 (60.8)
No	92 (31.0)	9 (17.7)	52(31.1)	31 (39.2)
Co-worker support				
Good co-worker support	277 (93.3)	51 (100)	154 (92.2)	72 (91.1)
Low co-worker support	20 (6.7)	0 (0)	13 (7.8)	7 (8.9)
Work amount				
Full-time	138 (46.5)	31 (60.8)	70 (41.9)	37 (46.8)
Part-time	121 (40.7)	15 (29.4)	81 (48.5)	25 (31.7)
Temp. work absence	38 (12.8)	5 (9.8)	16 (9.6)	15 (21.5)

Table 2. Associations between health, demographic, socio-economic and workplace factors and significant future work limitation in primary care consulters for osteoarthritis; comparing EWL with no EWL odds ratios with 95% confidence intervals

			Multivariate model within each domain		Multivariate model including all domains	
	OR*	95%CI†	OR	95%CI	OR	95%CI
Health factors						

For peer review only - http://bmjopen.bmj.com/site/about/guidelines.xhtml

Physical function	0.93	0.91, 0.96	0.94	0.90, 0.97	0.95	0.92, 0.97
Extent of pain						
No pain	1		1		-	
Some	1.67	0.14, 19.5	1.38	0.26, 7.340	-	
Widespread	5.58	0.48, 65.1	2.25	0.40, 12.7	-	
Comorbidity						
Low comorbidity (0-3)	1		1		-	
Multimorbidity (4 or more)	1.48	0.71, 3.10	0.66	0.24, 1.83	-	
Smoking						
Never	1		1		1	
Previously	1.42	0.63, 3.18	3.08	0.92, 10.2	1.52	0.51, 4.51
Currently	2.75	1.02, 7.38	6.85	1.48, 31.7	2.02	0.55, 7.40
Depression						
Non-case (0-7)	1		1		-	
Possible/probable case (8-21)	4.51	1.81, 11.3	1.93	0.44, 8.42	-	
Anxiety						
Non-case (0-7)	1		1		-	
Possible/probable case (8-21)	1.94	0.95, 3.97	0.62	0.15, 2.47	-	
Body mass Index						
Normal (20-24.9 kg m^2)	1		1		-	
Underweight (<20)	1.80	0.14, 23.4	0.52	0.01, 24.7	-	
Overweight (25-29.9 kg m^2)	1.13	0.39, 3.21	0.98	0.26, 3.62	-	
Obese (>30 kg m^2)	2.08	0.71, 6.10	0.76	0.16, 3.51	-	
Cognitive impairment						
No cognitive complaint	1		1		-	
Cognitive complaint	3.84	1.81, 8.18	3.28	0.84, 12.8	-	
Control of health						
Can control health	1		1		-	
Can't control health	1.56	0.38, 6.31	0.76	0.10, 5.81	-	
C- statistic			0.8641			
Socio-demographic						
Age						
	0.69	0.58, 0.82	0.67	0.56, 0.81	0.74	0.60, 0.91
Gender						
Male	1		1		-	
Female	1.82	0.89, 3.72	1.37	0.58, 3.22	-	
Educational attainment						
Further	1		1		-	
School only	1.38	0.56, 3.36	1.12	0.34, 3.64	-	
Occupational classification						
Managerial/Professional	1		1		-	
Intermediate	2.01	0.72, 5.63	2.27	0.64, 8.12	-	
Routine	1.94	0.84, 4.53	2.26	0.74, 6.90	-	
Adequacy of income						
Adequate	1		1		-	
Inadequate	1.97	0.94, 4.12	2.01	0.84, 4.81	-	
Live alone						
No	1		1		-	
Yes	0.48	0.17, 1.31	0.50	0.16, 1.56	-	
C-statistic			0.7636			
Workplace factors						
Work type						
Sedentary	1		1		-	
Standing	2.47	0.89, 6.88	2.32	0.60, 7.92	-	
Physical	0.85	0.33, 2.15	0.46	0.13, 1.59	-	
Heavy manual	4.24	0.79, 22.8	2.38	0.30, 18.7	-	
Work status						
Full-time	1		1		-	
Part-time	1.40	0.63, 3.10	1.07	0.40, 2.87	-	
Off work	2.85	0.94, 8.60	0.26	0.04, 1.91	-	
Physically demands of job						
Not physically demanding	1		1		-	
Physically demanding	3.18	1.50, 6.72	1.81	0.63, 5.21	-	
Flexible working						
Flexible	1		1		-	
Not flexible	1.36	0.65, 2.86	1.77	0.62, 5.04	-	
Use of aids and appliances						
Yes	1		1		-	
No	1.64	0.80, 3.37	1.06	0.43, 2.65	-	
Opportunities to retrain						
Yes	1		1		-	
No	3.01	1.29, 7.05	1.99	0.72, 5.45	-	
Work satisfaction						

For peer review only

Satisfied	1		1		1	
Dissatisfied	3.69	1.43, 9.49	4.78	1.57, 14.6	2.08	0.66, 6.51
C-statistic			0.7785		0.8418	

*Odds ratio; † 95 percent confidence interval;

Figure 1. Flow diagram of participants for the longitudinal analysis

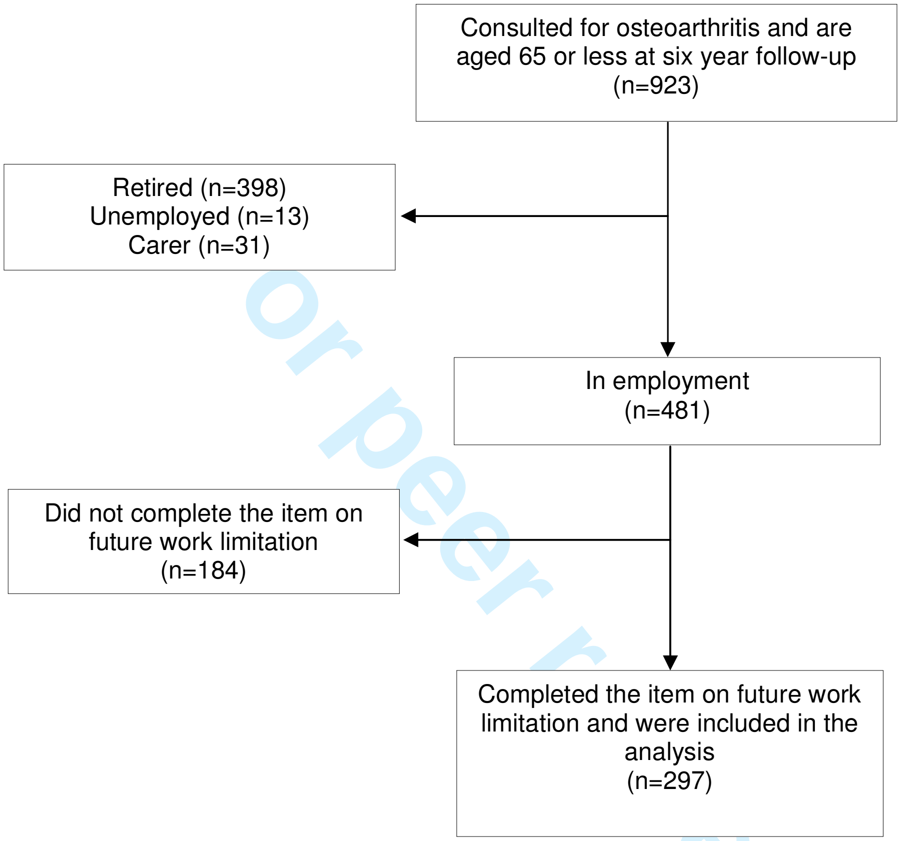

For peer review only - http://bmjopen.bmj.com/site/about/guidelines.xhtml

STROBE Statement—Checklist of items that should be included in reports of ***cohort studies***

	Item No	Recommendation
**Title and abstract**	1	(*a*) Indicate the study's design with a commonly used term in the title or the abstract<br><br>The title of the study is: "Anticipated significant work limitation in primary care consulters with osteoarthritis: a prospective cohort study"
		(*b*) Provide in the abstract an informative and balanced summary of what was done and what was found<br><br>The abstract summarises the methods and key results
**Introduction**		
Background/rationale	2	Explain the scientific background and rationale for the investigation being reported<br><br>The scientific background and rationale for the investigation are reported in the introduction (see page 3).<br>"Osteoarthritis is the most common joint condition in adults and globally is the fastest increasing major heath condition [1]. It is a common reason for primary care consultation (one in twenty consultations in adults aged between 45 and 65 per year is for osteoarthritis [2]) and is recognised as one of the leading and rapidly growing causes of disability [3]. Its most disabling manifestation (joint pain) is strongly associated with ageing [4] and with the commonest forms of disability [5-9].<br>Work restriction is one form of disability that will become more important for those with osteoarthritis and joint pain because increases in state pension age in many developed countries means that most adults can expect a need to continue working at older ages than before [10]. Normal retirement age in North America and Europe has increased, and is expected to rise further to 69 and beyond [11]. However the extent to which participation in work will be limited by health-related problems, resulting in significant work limitation in terms of absenteeism and presenteeism (remaining in work but with limitation and reduced productivity) is unclear [12]. The increasing prevalence of chronic health conditions, especially osteoarthritis, in persons near to retirement age raises questions about viability of attempts to extend working life. Several studies of expectations of future work loss are predictive of future work outcomes [13,14]. Identifying the prevalence and predictors of expected work limitations (EWL) in this group of patients, particularly those that are amenable to change, will inform management and possible preventative strategies for future work limitation."
Objectives	3	State specific objectives, including any prespecified hypotheses<br><br>Objectives of the study are stated in the last paragraph of the Introduction on page 3-4.

For peer review only - http://bmjopen.bmj.com/site/about/guidelines.xhtml

		The aim of this study was to estimate the proportion of working age adults with osteoarthritis who predict that joint pain would limit their work or stop them working (i.e. EWL) prior to a possible future pension age of 69. In addition health, socio-demographic or workplace factors associated with EWL, especially those amenable to change, were explored, to identify potential targets to manage and prevent EWL [15-17].
**Methods**		
Study design	4	Present key elements of study design early in the paper<br><br>Key elements of study design are presented in the first sentence of the Study Design paragraph in the Method section.<br>"The North Staffordshire Osteoarthritis project (NorStOP) is a population-based prospective cohort study."
Setting	5	Describe the setting, locations, and relevant dates, including periods of recruitment, exposure, follow-up, and data collection<br><br>Please see the Study Design paragraph in the Method section, as quoted in 4.
Participants	6	(*a*) Give the eligibility criteria, and the sources and methods of selection of participants. Describe methods of follow-up<br><br>Same as above. Please also see Figure 1 and the first paragraph of the Results section
		(*b*) For matched studies, give matching criteria and number of exposed and unexposed<br><br>Not applicable
Variables	7	Clearly define all outcomes, exposures, predictors, potential confounders, and effect modifiers. Give diagnostic criteria, if applicable<br><br>Please see the sections spanning from page 5 to page 8 (Identification of osteoarthritis to the end of workplace factors).
Data sources/ measurement	8*	For each variable of interest, give sources of data and details of methods of assessment (measurement). Describe comparability of assessment methods if there is more than one group<br><br>Same as 7.
Bias	9	Describe any efforts to address potential sources of bias<br><br>Additional analysis was undertaken to examine the potential impact of missing data (non-completion of the outcome question) (see paragraph 2 of the methods).

For peer review only

Study size	10	Explain how the study size was arrived at<br>The derivation of the study sample is explained in the second paragraph of the methods section<br>The study sample was based on those who (i) consulted for osteoarthritis during the study period (starting 18 months before the baseline questionnaire was administered, and continuing through the time of the final follow-up questionnaire (i.e. from 2000 to 2008)), (ii) were of working age (less than 65 years old) and in employment at the six year follow-up and (iii) completed the item on expected work limitation prior to 69 years old (EWL) at six year follow-up.
Quantitative variables	11	Explain how quantitative variables were handled in the analyses. If applicable, describe which groupings were chosen and why<br>This is outlined in the Statistical Analysis section on pages 8.
Statistical methods	12	(*a*) Describe all statistical methods, including those used to control for confounding<br>This is outlined in the Statistical Analysis section on pages 8
		(*b*) Describe any methods used to examine subgroups and interactions<br>Not applicable
		(*c*) Explain how missing data were addressed<br>Patients were included only if they had complete data
		(*d*) If applicable, explain how loss to follow-up was addressed<br>The potential bias due to loss of participants is summarised in the paper
		(*e*) Describe any sensitivity analyses<br>Same as 12(d)
**Results**		
Participants	13*	(a) Report numbers of individuals at each stage of study—eg numbers potentially eligible, examined for eligibility, confirmed eligible, included in the study, completing follow-up, and analysed<br>These are described in the text and if further illustrated in figure 1.
		(b) Give reasons for non-participation at each stage<br>The reasons for non-participation are provided at each stage
		(c) Consider use of a flow diagram

For peer review only

For peer review only - http://bmjopen.bmj.com/site/about/guidelines.xhtml

		See Figure 1
Descriptive data	14*	(a) Give characteristics of study participants (eg demographic, clinical, social) and information on exposures and potential confounders<br><br>See the first paragraph of the results and Table 1
		(b) Indicate number of participants with missing data for each variable of interest<br><br>The analysis has been done in those with complete data
		(c) Summarise follow-up time (eg, average and total amount)<br><br>Data included in this study has been collected over 6 years. Please see methods section.
Outcome data	15*	Report numbers of outcome events or summary measures over time<br><br>Results in the text and tables adhere to these guidelines.
Main results	16	(*a*) Give unadjusted estimates and, if applicable, confounder-adjusted estimates and their precision (eg, 95% confidence interval). Make clear which confounders were adjusted for and why they were included<br><br>Results in the text and tables adhere to these guidelines. See also Table 2
		(*b*) Report category boundaries when continuous variables were categorized<br><br>Same as 16(a)
		(*c*) If relevant, consider translating estimates of relative risk into absolute risk for a meaningful time period<br><br>Not applicable.
Other analyses	17	Report other analyses done—eg analyses of subgroups and interactions, and sensitivity analyses<br><br>All analyses are fully reported in the statistical methods section
**Discussion**		
Key results	18	Summarise key results with reference to study objectives<br><br>This is done in the first paragraph (pages 11-12)
Limitations	19	Discuss limitations of the study, taking into account sources of potential bias or imprecision. Discuss both direction and magnitude of any potential bias

For peer review only - http://bmjopen.bmj.com/site/about/guidelines.xhtml
For peer review only

		This is done in the strengths and limitations section of the discussion (pages 12-13)
Interpretation	20	Give a cautious overall interpretation of results considering objectives, limitations, multiplicity of analyses, results from similar studies, and other relevant evidence<br><br>This is done in the comparison with existing literature section of the discussion (pages 10-15)
Generalisability	21	Discuss the generalisability (external validity) of the study results<br><br>This is done in the strengths and limitations section of the discussion (pages 12-13)
**Other information**		
Funding	22	Give the source of funding and the role of the funders for the present study and, if applicable, for the original study on which the present article is based<br><br>This information is disclosed at the end of the manuscript

*Give information separately for exposed and unexposed groups.

Note: An Explanation and Elaboration article discusses each checklist item and gives methodological background and published examples of transparent reporting. The STROBE checklist is best used in conjunction with this article (freely available on the Web sites of PLoS Medicine at http://www.plosmedicine.org/, Annals of Internal Medicine at http://www.annals.org/, and Epidemiology at http://www.epidem.com/). Information on the STROBE Initiative is available at http://www.strobe-statement.org.

For peer review only

For peer review only - http://bmjopen.bmj.com/site/about/guidelines.xhtml

BMJ Open

Anticipated significant work limitation in primary care consulters with osteoarthritis: a prospective cohort study

Journal:	*BMJ Open*
Manuscript ID:	bmjopen-2014-005221.R1
Article Type:	Research
Date Submitted by the Author:	15-Jul-2014
Complete List of Authors:	Wilkie, Ross; Keele University, Research Institute for Primary Care and Health Sciences<br>Phillipson, Christopher; University of Manchester, School of Social Sciences<br>Hay, Elaine; Keele University, Research Institute for Primary Care & Health Sciences<br>Pransky, Glenn; Liberty Mutual Research Institute,
**Primary Subject Heading**:	General practice / Family practice
Secondary Subject Heading:	Epidemiology, Rheumatology, Occupational and environmental medicine
Keywords:	PRIMARY CARE, EPIDEMIOLOGY, OCCUPATIONAL & INDUSTRIAL MEDICINE, Musculoskeletal disorders < ORTHOPAEDIC & TRAUMA SURGERY, RHEUMATOLOGY

SCHOLARONE™
Manuscripts

Anticipated significant work limitation in primary care consulters with osteoarthritis: a prospective cohort study

Ross Wilkie[1], Chris Phillipson[2], Elaine M Hay[1], Glenn Pransky[3]

[1]Arthritis Research UK Primary Care Centre, Primary Care Sciences, Keele University, Keele, Staffordshire, United Kingdom, ST5 5BG.
[2] School of Social Sciences, The University of Manchester, Manchester, United Kingdom, M13 9PL
[3] Center for Disability Research, Liberty Mutual Research Institute, 71 Frankland Rd., Hopkinton MA 01748. United States.

Address for reprint requests and for corresponding author
Dr. Ross Wilkie
Arthritis Research UK Primary Care Centre, Primary Care Sciences, Keele University, Keele, Staffordshire, United Kingdom, ST5 5BG.
Email: r.wilkie@keele.ac.uk
Tel: +44 1782 734845
Fax: +44 1782 583911

Short title: Expected work loss in osteoarthritis consulters

Key indexing terms: osteoarthritis, work disability, epidemiology, primary health care, joint pain

For peer review only

For peer review only - http://bmjopen.bmj.com/site/about/guidelines.xhtml

Abstract

Objective: To describe the prevalence of expected work limitations (EWL) prior to future retirement age in osteoarthritis consulters, and the associated health, socio-demographic and workplace factors.

Design: Population-based prospective cohort study.

Setting: General practices in Staffordshire, England.

Participants: 297 working adults aged 50 to 65, who had consulted primary care for osteoarthritis.

Outcome: EWL was defined using a single question, "Do you think joint pain will limit your ability to work before you reach 69 years old".

Results: 51 (17.2%) indicated that joint pain would not limit their ability to work until 69, 79 (26.6%) indicated EWL and 167 (56.2%) did not know if joint pain would limit work before 69. In bivariate analysis, physical function (Odds ratio 0.93; 95% confidence interval 0.91, 0.96), depression (4.51; 1.81, 11.3), cognitive complaint (3.84; 1.81, 8.18), current smoker (2.75; (1.02, 7.38), age (0.69; 0.58, 0.82), physically-demanding job (3.18; 1.50, 6.72), no opportunities to retrain (3.01; 1.29, 7.05) and work dissatisfaction (3.69; 1.43, 9.49) were associated with EWL. The final multivariate model, included physical function and age.

For peer review only

Conclusions: Only one in five osteoarthritis consulters expected that joint pain would not limit their work participation before 69 years old. Given the expectation for people to work until they're older, the results highlight the increasing need for clinicians to include work participation in their consultation and implement strategies to address work loss/limitation. Targeting pain related functional limitation and effective communication with employers to manage workplace issues could reduce EWL.

Article summary

- Osteoarthritis is a common reason for primary care consultation and joint pain is strongly associated with ageing and disability. Loss of work participation is one form of disability that will become more important as adults work to older ages, due to rising state pension age, and have greater financial needs resulting from inadequate retirement resources.
- The aim of this study was to estimate the proportion of working age adults with osteoarthritis who predict that joint pain would limit their work or stop them working prior to a possible future pension age of 69 (i.e. EWL) and identify health, socio-demographic and workplace factors associated with EWL.

Key messages

- Only one in five consulters for osteoarthritis expected that joint pain would not limit their work participation before 69 years old.
- Low physical function, being a current smoker, depression, and several workplace factors - physically-demanding job, work dissatisfaction, and poor co-worker support – were associated with anticipated early work limitation.

Strengths and Limitations

For peer review only - http://bmjopen.bmj.com/site/about/guidelines.xhtml

- The sample is representative of primary care consulters with physician diagnosed osteoarthritis.
- The outcome is based on individual's expectations but this can be highly predictive of future work loss/limitation and drive consultation for health care.

For peer review only

Introduction

Osteoarthritis is the most common joint condition in adults and globally is the fastest increasing major heath condition [1]. It is a common reason for primary care consultation (one in twenty consultations in adults aged between 45 and 65 per year is for osteoarthritis [2]) and is recognised as one of the leading and rapidly growing causes of disability [3]. Its most disabling manifestation (joint pain) is strongly associated with ageing [4] and with the commonest forms of disability [5-9].

Work restriction is one form of disability that will become more important for those with osteoarthritis and joint pain because increases in state pension age in many developed countries means that most adults can expect a need to continue working at older ages than before [10]. Normal retirement age in North America and Europe has increased, and is expected to rise further to 69 and beyond [11]. However the extent to which participation in work will be limited by health-related problems, resulting in significant work limitation in terms of absenteeism and presenteeism (remaining in work but with limitation and reduced productivity) is unclear [12]. The increasing prevalence of chronic health conditions, especially osteoarthritis, in persons near to retirement age raises questions about viability of attempts to extend working life. Several studies of expectations of future work loss are

For peer review only - http://bmjopen.bmj.com/site/about/guidelines.xhtml

predictive of future work outcomes [13,14]. Identifying the prevalence and predictors of expected work limitations (EWL) in this group of patients, particularly those that are amenable to change, will inform management and possible preventative strategies for future work limitation. The aim of this study was to estimate the proportion of working age adults with osteoarthritis who predict that joint pain would limit their work or stop them working (i.e. EWL) prior to a possible future pension age of 69. In addition health, socio-demographic or workplace factors associated with EWL, especially those amenable to change, were explored, to identify potential targets to manage and prevent EWL [15-17].

Method

Study population

The North Staffordshire Osteoarthritis project (NorStOP) is a population-based prospective cohort study. The NorStOP sampling frame comprised all individuals aged 50 years and over who were registered to receive care from one of six general practices in North Staffordshire, England, United Kingdom (UK). In 2002, adults aged who gave their written consent for medical record review were followed up over 6 years for consultation to primary care. They were also mailed questionnaires at three and six years; reminders were sent at two and four weeks after the initial mailing. The North Staffordshire Local Research Ethics Committee approved this study.

Analyses for this paper included those who (i) consulted for osteoarthritis during the study period (starting 18 months before the baseline questionnaire was administered, and continuing through the time of the final follow-up questionnaire (i.e. from 2000 to 2008)), (ii) were of working age (less than 65 years old) and in employment at the six year follow-up and (iii) completed the item on expected work limitation prior to 69 years old (EWL) at six year follow-up. Over the study period there were 923 adults who had consulted for osteoarthritis

For peer review only - http://bmjopen.bmj.com/site/about/guidelines.xhtml

and were of working age at 6 year follow-up. Of this group 398 had retired before state retirement age, 13 were unemployed, 31 were homemakers, leaving 481 who were in employment and thus eligible for the study. Of these, 184 did not complete the item on future work limitation, leaving complete data for 297 participants (adjusted response 61.7%) (Figure 1). Compared to those subjects who had consulted for osteoarthritis but did not complete the item on future work limitation questionnaire (n=184) those included in the analysis (n=297) were more likely to be female (p=0.02) and have an adequate income p=0.052) but no more likely to be older (p=0.19), have better physical (p=0.91) or mental health (p=0.21) or have gone onto further education (p=0.52).

Identification of osteoarthritis

General practitioners in the study used the Read system to code all reasons for clinical encounters in primary care consultations [18]. The Read codes cross-map to ICD9/ICD-10 (for diseases). Morbidity data (i.e. symptoms and diseases) in this system are grouped under 19 main Read chapters. Data collected at the second hierarchical level or above was used to identify diagnostic groups, and these were aggregated starting 18 months before the baseline questionnaire was administered, and continuing through the time of the final follow-up questionnaire. Individuals were defined as having osteoarthritis if they had at least one consultation during this period primarily for osteoarthritis based on Read codes (N05 category) for primary care consultations [18]. As osteoarthritis is a long-standing, gradually progressive chronic condition, it was assumed that a clinician-established diagnosis at some point during the study period implied that OA was likely present at least to some degree during the entire period of observation.

For peer review only - http://bmjopen.bmj.com/site/about/guidelines.xhtml

Outcome measure

Expected work limitation (EWL) was defined using a single question at six-year follow-up, “do you think joint pain will limit your ability to work before you reach 69 years old” (will limit or stop me/ don’t know/ won’t limit).

Independent factors

Health factors were measured across the six year study period, socio-demographic factors at three year follow-up and workplace factors, were measured retrospectively at six year follow-up (Table 1).

Health Factors

Physical function was measured at each time point using the physical functioning scale of the Medical Outcomes Study Short Form-36; score range: 0-100, higher scores indicating better function [19]. Items measured the limitation in the individual’s capacity to complete basic tasks such as lifting and walking. Scores at each time point were highly correlated (i.e. between baseline and three year follow-up r=0.71; between three and six year follow-up r=0.75). Physical function score at three year follow-up (the middle point of data collection) was used in this analysis. The extent of musculoskeletal pain was measured by responders shading painful areas (0-44) on a full body diagram (front and back views). These methods to determine the location and extent of pain are commonly used in population based studies of pain, and have been shown to be valid and reliable [20]. Using these pain drawings, participants were classified into one of three groups (none, some and widespread). The widespread group were those participants that satisfied the criteria for widespread pain included in the American College of Rheumatology 1990 criteria for fibromyalgia [21] at baseline, three or six years. These criteria require pain to be present above and below the waist, in the right and left hand sides of the body and in the axial skeleton; remaining

For peer review only - http://bmjopen.bmj.com/site/about/guidelines.xhtml

participants who reported pain at any time point that did not satisfy the criteria for widespread were classified as having "some pain" and those participants who did not report pain at all were classified as "no pain".

Comorbidity was identified using Read diagnostic codes from primary care consultations. Multimorbidity was defined as 4 or more comorbidities (different major diagnostic groups) in the 2 years prior to baseline, between baseline and 3 year follow-up or between three and 6 year follow-up [22]. Anxiety and depression during the previous week were measured using the Hospital Anxiety and Depression Scale (HADS) - raw scores categorised individuals as non-cases (0-7) or possible/probable cases (8-21); depression was defined as possible or probable case at any of the three time points [23]. Self-reported height and weight were categorised into standard BMI groups (i) normal weight (BMI 20-24.9kgm^{-2}), (ii) underweight (BMI <20kgm^{-2}), (iii) overweight (BMI 25 – 29.9 kgm^{-2}) and (iv) obese (BMI ≥30kgm^{-2}). Cognitive complaint was measured using the Alertness Behaviour Subscale of the Sickness Impact Profile [24]. This scale has 10 items that ask about alertness and ability to concentrate. Each item was scored as 0 (no cognitive complaint) or 1 (cognitive complaint) with raw additive scores categorised to indicate "no cognitive complaint" (score of 0) and "cognitive complaint" (score >0); individuals were identified as having cognitive complaint if they had a score >0 at any of the three time points. Perceived control of health was measured using a single item at baseline (There is a lot I can do to control my health yes/no). Participants were also asked to report their smoking status at baseline (current, previous, never).

Socio-demographic Factors

Demographic and socio-economic details included age, gender, educational attainment (those who finished their education on leaving school; those who went onto further education such

For peer review only - http://bmjopen.bmj.com/site/about/guidelines.xhtml

as college or university), occupational class (managerial or professional (chief executive, professor), intermediate (e.g. paramedics, technicians), routine (machine operator, childcare worker), adequacy of income (Thinking about the cost of living as it affects you, which of these descriptions best describes your situation; adequate/inadequate) and living status (live alone/live with others).

Workplace Factors

Single items on the 6-year follow-up questionnaire measured workplace characteristics. Items included work type (Which of the following statements best describes the work that you do in your current job; sedentary occupation/standing occupation/physical work/heavy manual work), work status (current employment status: full-time/part-time/temporarily off work (e.g. due to sickness), physically demanding employment (Thinking over the last 30 days: Is your work physically demanding; not physically demanding/physically demanding), flexible working (My hours of work are flexible; flexible/not flexible), co-worker support (My work colleagues are supportive: good/low), work satisfaction (How satisfied are you with your current job satisfaction/dissatisfied), opportunities to retrain (There are opportunities to retrain and develop my skills; yes/no) and able to use aids and appliances and adapt the workplace (I can use aids and appliances to help me do my job or adapt my work; yes/no).

Statistical analysis

First, the distribution of health, socio-demographic and workplace factors was examined by prediction of the ability to work until 69 with differences tested for significance using Chi-square or Kruskall Wallis tests where appropriate. Regression analyses then focused on identifying factors associated with significant future work loss (will be limited in ability to work, or will be unable to work until age 69 because of joint pain (i.e. EWL)); compared to no expected work loss. Bivariate and multivariate logistic regression models were

For peer review only - http://bmjopen.bmj.com/site/about/guidelines.xhtml

constructed to examine the relationship between each health, socio-demographic and workplace factor and EWL. Following bivariate analysis, multivariate models included all of the factors within each category (i.e. all health factors in one model, all socio-demographic in the second model and all of the workplace factors in the third model for each outcome). Factors independently associated with EWL within each category were then included in a final multivariate model. To evaluate model fit, concordance indexes (C-statistic) were calculated for each model. A C-statistic of 0.50 indicates the predictive ability of a model to be no better than chance, 0.7 indicates reasonable, 0.8 indicates high and 1.0 indicates perfect predictive ability [25].

Stata version 11 was used for all analyses. The results of the analyses are presented as odds ratios (OR) with 95% confidence intervals (CI). For the regression analyses the "won't be limited" group was classified as the referent category.

Results

Of the 297 consulters for osteoarthritis included in the analysis, 51 (17.2%) indicated that joint pain would not limit their ability to work until 69 years, 79 (26.6%) indicated that joint pain would limit or stop them working before 69 years (EWL) and 167 (56.2%) indicated that they did not know if they would have EWL before 69 years. Those who indicated EWL (median age 53 years) were younger than those who did not know (median age 54 years) or did not predict EWL (median age 57; p=0.0001) (Table 2). Women were more likely to indicate that they did not know or would have EWL (p=0.01). There was no significant difference among the three groups for educational attainment (p=0.44) or occupational classification (p=0.10). Notably all responders with low co-worker support predicted developing EWL before age 69 years.

For peer review only - http://bmjopen.bmj.com/site/about/guidelines.xhtml

Table 3 shows the results of the regression analysis comparing anticipated EWL to no EWL. In the bivariate analysis, health, socio-economic and workplace factors were associated with EWL before age 69 years. Of the health factors, depression (odds ratio 4.51; 95% confidence interval 1.81, 11.3), cognitive complaint (3.84; 1.81, 8.18) and being a current smoker (2.75; (1.02, 7.38) were associated with EWL onset prior to 69 years. Increasing physical function (0.93; 0.91, 0.96) was protective against EWL onset prior to 69 years. In the multivariate analysis of health factors only increasing physical function (0.94; 0.90, 0.97) was associated with EWL onset before 69 years. Of the socio-economic factors, only age (0.69; 0.58, 0.82) was associated with EWL. Of the workplace factors, a physical demanding job (3.18; 1.50, 6.72), no opportunities to retrain (3.01; 1.29, 7.05) and work dissatisfaction (3.69; 1.43, 9.49) were associated with EWL. In the multivariate analysis of workplace factors, a physically demanding job and work satisfaction were independently associated with EWL. In the final multivariate model, combining significant factors from the health, socio-economic and workplace factors, the association with work dissatisfaction attenuated to insignificance (adjusted odds ratio 2.08; 95% confidence interval 0.66, 6.51). Physical function (0.95; 0.92, 0.97) and age (0.74; 0.60, 0.91) remained independently associated with EWL; for every 1 point increase in physical function the odds of EWL increased by 7% and for every 1 year increase in age, the odds of EWL decreased by 45%. The model fit of the final model was 0.8418.

Discussion

Principal findings

For peer review only - http://bmjopen.bmj.com/site/about/guidelines.xhtml

For peer review only

This study is the first to examine the factors associated with an expectation of having significant future work limitation (EWL) for employed patients who consult primary care physicians for osteoarthritis. Physical function, being a current smoker, depression, and several workplace factors - physically-demanding job, work dissatisfaction, and poor co-worker support – were associated with anticipated EWL. Only one in five osteoarthritis consulters (17.2%) indicated that joint pain would not limit their ability to work until age 69. Given the high and increasing prevalence of osteoarthritis, the number of consultations for this disorder, and the anticipated extension of working lives due to changes in retirement policy, these results raise significant concerns, while suggesting potential areas for intervention.

The proportion of osteoarthritis cases who expected work limitation was higher compared to previous studies. Using a national US sample of all workers, Theis and Murphy found that only a third of those with physician-diagnosed arthritis reported work limitations [15]. Unlike the current study, their sample was not restricted to employed persons, and did not ask about expected ability to work in the future. Studies of rheumatoid arthritis patients have found that premature work loss in persons in a similar age group is common, affecting up to 90% of persons with this diagnosis, and leads to significant economic and social consequences [26]. Our finding of a similar high rate of expected work loss in persons with OA suggests that the societal impact in this much larger group will be significant as well.

Physical function was highly protective against EWL. Reduced physical function has been found to be associated with work limitations in persons with any type of arthritis [15], musculoskeletal disorders in general [27] or those with a specific arthritis diagnosis [28,29]. This indicates an important mismatch between individual capabilities and work demands,

For peer review only - http://bmjopen.bmj.com/site/about/guidelines.xhtml

that is more important than pain by itself. High work physical demands have long been recognized as an important risk factor for subsequent work disability in rheumatoid arthritis [27]. Some have suggested that control over work demands might be more important than the absolute level of work demands, but these studies did not evaluate the level of job physical demands [30]. Work dissatisfaction was also significant, although this problem was reported in relatively few respondents. There may be several dimensions of work dissatisfaction – not only being dissatisfied at work, but also a preference to be at home instead [30]. Similarly, lack of co-worker support was a problem in only a relatively small number of persons, but all who reported low co-worker support expected to have EWL. All of those who reported low co-worker support also indicated at least one significant health problem (i.e. widespread pain, multimorbidity, depression or anxiety). The importance of co-worker support in those returning to work after injury or illness, across a range of conditions, has recently been recognized [31], and low co-worker support has been associated with greater job strain and work loss in workers with arthritis [32,33]. Alternatively, this finding may be less related to co-worker support than the particular nature of their job, such as working on their own, or job types that have little involvement with others. If trends in the arrangements of jobs in the future means that more older adults with osteoarthritis are working on their own the significance of low co-worker support will increase.

We did not see the effects of low education (e.g. [15]) or comorbidities reported by others. Education may not have a large impact compared to actual job physical demands. Flexible work was not a factor, although it has been identified in other studies as a significant

For peer review only - http://bmjopen.bmj.com/site/about/guidelines.xhtml

predictor of staying at work with RA [34]. This difference may be due to the less specific nature of the question in this study, compared with other investigations.

The majority of participants (52.6%) could not predict whether pain would limit or stop their ability to work until the new retirement age. In additional analysis, a forward stepwise logistic regression model of health, socio-demographic and workplace factors was constructed to identify which factors were associated with being unable to predict whether pain would limit the ability to work. Lower age, being in part time work and being unable to use aids and appliances were the factors significantly associated with being unable to predict. Their responses may be due to some uncertainty about the future progression of their condition, or about whether or not employment until age 69 would be required. Notably the greater likelihood for women to indicate "don't know" was explained by these factors. The role of aids and appliances again indicates the importance of the workplace to allow individuals to self-manage their pain to optimise performance.

Strengths and weaknesses

The strength of this study's longitudinal design enables prospective identification of factors associated with EWL in a clinically relevant primary care population. The sample is representative of primary care consulters with physician diagnosed osteoarthritis, relevant to primary care practices. Other studies have been limited to patients from rheumatology practices or rehabilitation clinics, a less representative sample of osteoarthritis patients.

There are limitations to this study. A range of factors have been identified as potentially linked to future employment loss in osteoarthritis, and some were not included in the information collected for this study, such as extent of joint involvement, success of current

For peer review only - http://bmjopen.bmj.com/site/about/guidelines.xhtml

coping strategies, opportunities for part-time work or retirement, importance of work role, social support outside of the workplace, and extent of workplace accommodations specific to their osteoarthritic condition [16]. In addition, overall pain levels, illness perceptions and health beliefs have not been included, which may have considerable impact on predicted work outcomes [35]. We chose to measure extent of pain rather than pain level, as we have previously found extent of pain to be associated with poor work outcomes and this may be more reflective of total arthritis impact on physical function than pain intensity [36]. However, clinical measures may not be as important in predicting work disability, compared to measure of function, psychosocial and workplace factors [34]. The C-statistics for the health (0.8641), socio-demographic (0.7636), workplace (0.7785) and final (0.8418) models indicate a reasonable or high ability to predict EWL. The factors included here are those thought to be most important in maintaining employment in chronic musculoskeletal conditions. Data on most variables was by self-report, but validated instruments were used to measure anxiety, depression and pain extent. The outcome is based on individual's expectations but this can be highly predictive of future work loss/limitation and drive consultation for health care [15-17]. We did not have radiographic or detailed information on the extent of OA, but the intention of the study was to describe a typical, heterogeneous group of patients with OA as seen in primary care practice. Measurement of predictors at three time points may not reflect changes in these factors during follow-up. Workplace factors were measured only at six year follow-up, and may be out of sequence with health factors, but give a sense of current workplace status at the same time that the question on anticipated EWL was asked. As with any cohort study, non-completion of the items may affect estimates, however based on comparisons between those included in the analysis and those who did not complete the EWL item, such effects are likely to be small. The area

For peer review only - http://bmjopen.bmj.com/site/about/guidelines.xhtml

covered by the study is more deprived on health, education, and employment, but with fewer barriers to housing and services, than England as a whole, but again the potential effect of this on estimates will be small.

Clinical implications

With current disease prevalence and economic trends, and increasing prevalence of osteoarthritis, clinicians will undoubtedly have more consultations with persons concerned about their ability to stay at work [37]. Studies in rheumatoid arthritis suggest that a proactive approach – identification and intervention before work loss has occurred – can be effective in preventing subsequent work loss [38]. In this study, health problems were present well in advance of when EWL was measured. For example, the high correlation between physical function scores at baseline and three years (0.71) indicates that low physical function was experienced six years before EWL was measured. This indicates that there is a lot of time to intervene with available strategies [39]. Certain types of coping strategies may be more effective than others in maintaining employment in persons with arthritis, especially anticipating challenges in the workplace and formulating strategies to deal with them in advance [40]. Given the importance of osteoarthritis-related physical limitations at work as a predictor of EWL, a positive screening question for these limitations should lead to a more in-depth discussion about accommodations and employment options, and perhaps a referral to a vocational expert [41,42]. The negative association with inadequate co-worker support suggests that employer engagement in creating a supportive work environment will also be a major factor in work retention for these patients [33]. Addressing the needs of employed osteoarthritis patients within the context of a broader psychosocial model of disease ensures a broader and more relevant perspective on the

For peer review only - http://bmjopen.bmj.com/site/about/guidelines.xhtml

causes and prevention opportunities for subsequent work loss [17]. There are still many unanswered questions about the optimal nature, timing and duration of interventions designed to maintain employment, and how to engage treating clinicians as positive contributors; thus further studies will be needed in order to understand how best to address these problems [43,44].

Conclusion

This observational study suggests 4 out of 5 consulters to primary care with osteoarthritis expect their joint pain to limit their work participation prior to future pension age, and that younger age and greater arthritis-related physical limitations are the main factors associated with this expected outcome. Given the expectation for people to work until they are older with osteoarthritis, the results highlight the increasing need for clinicians to include work participation in their consultation and implement strategies to prevent work limitation. Targeting pain related functional limitation and effective communication with employers to manage workplace issues could reduce the expectation of future work limitation.

For peer review only

For peer review only - http://bmjopen.bmj.com/site/about/guidelines.xhtml

ACKNOWLEDGEMENTS

We would like to thank the administrative and health informatics staff at Keele University's Arthritis Research UK Primary Care Centre and the doctors and staff of the participating general practices.

FINANCIAL DISCLOSURE: This study is supported financially by the Medical Research Council, UK (grant code: G9900220) and by the North Staffordshire Primary Care R&D Consortium.

CONTRIBUTORSHIP STATEMENT

RW is the principal investigator of the project, responsible for the conception and design of the study. He was involved in the data analysis and led the drafting and final editing of the paper. CP and EM are guarantors and advised on the study design. GP is co-investigator of the project. He led on the data analysis and contributed to the drafting and final editing of the manuscript. All authors contributed to the final version of the paper.

DATA SHARING: The Arthritis Research UK Primary Care Research Centre has established data sharing arrangements to support joint publications and other research collaborations. Applications for access to anonymized data from our research databases are reviewed by the Centre's Data Custodian and Academic Proposal (DCAP) Committee and a decision regarding access to the data is made subject to the NRES ethical approval first provided for the study and to new analysis being proposed. Further information on our data sharing procedures can be found on the Centre's website (http://www.keele.ac.uk/pchs/publications/datasharingresources/) or by emailing the Centre's data manager (data-sharing-pcs@cphc.keele.ac.uk).

CONFLICTS OF INTEREST: The authors declare no conflicts of interest

For peer review only

FUNDING: The NorStOP study was funded by a programme Grant from the MRC UK (grant code G9900220)

REFERENCES

1. Murray CJ, Vos T, Lozano R, et al. Disability-adjusted life years (DALYs) for 291 diseases and injuries in 21 regions, 1990-2010: a systematic analysis for the Global Burden of Disease Study 2010. Lancet 2012;380(9859):2197-223.

2. Jordan K, Clarke AM, Symmons DP, et al. Measuring disease prevalence: a comparison of musculoskeletal disease using four general practice consultation databases. Br J Gen Pract 2007;57:7-14.

3. Arden N, Nevitt MC. Osteoarthritis: epidemiology. Best Pract Res Clin Rheumatol 2006;20:3-25.

4. Doherty M. Risk factors for progression of knee osteoarthritis. Lancet 2001;358:775-6.

5. Jordan JM, Luta G, Renner JB, et al. Self-reported functional status in osteoarthritis of the knee in a rural southern community: the role of sociodemographic factors, obesity, and knee pain. Arthritis Care Res 1996;9:273-8.

6. Jinks C, Jordan K, Croft P. Osteoarthritis as a public health problem: the impact of developing knee pain on physical function in adults living in the community: (KNEST 3). Rheumatology 2007;46(5):877-81.

7. Felson DT, Lawrence RC, Dieppe PA, et al. Osteoarthritis: new insights. Part 1: the disease and its risk factors. Ann Intern Med 2000;133:635-46.

8. Wilkie R, Peat G, Thomas E, et al. Factors associated with restricted mobility outside the home in community-dwelling adults aged 50 years and over with knee pain – an example of use of the International Classification of Functioning to investigate participation restriction. Arthritis Rheum 2007;57(8):1381-9.

For peer review only - http://bmjopen.bmj.com/site/about/guidelines.xhtml

9. Odding E, Valkenburg HA, Stam HJ, et al. Determinants of locomotor disability in people aged 55 years and over: The Rotterdam study. Eur J Epidemiol 2002;17:1033-41.

10. Helman R, Copeland C, VanDerhei J. The 2012 Retirement Confidence Survey: job insecurity, debt weigh on retirement confidence, savings. EBRI Issue Brief 2012;369:5-32,1.

11. Organisation for Economic Co-operation and Development. OECD Pension Outlook 2012. http://www.oecd.org/pensions/private-pensions/50560110.pdf (accessed August 2013).

12. Bieleman HJ, Bierma-Zeinstra SM, Oosterveld FG, et al. The effect of osteoarthritis of the hip or knee on work participation. J Rheumatol 2011;38(9):1835-43.

13. Fadyl J, McPherson K. Return to work after injury: a review of evidence regarding expectations and injury perceptions, and their influence on outcome. J Occup Rehabil. 2008;18(4):362-74.

14. Reme SE, Hagen EM, Eriksen HR. Expectations, perceptions, and physiotherapy predict prolonged sick leave in subacute low back pain. BMC Musculoskelet Disord 2009;10:139. 15. Theis KA, Murphy L, Hootman JM, et al. Prevalence and correlates of arthritis-attributable work limitation in the US population among persons ages 18-64: 2002 National Health Interview Survey Data. Arthritis Rheum 2007;57(3):355-63.

16. Hoving JL, van Zwieten MC, van der Meer M, Sluiter JK, Frings-Dresen MH. Work participation and arthritis: a systematic overview of challenges, adaptations and opportunities for interventions. Rheumatology 2013;52(7):1254-64

17. Wilkie R, Pransky G. Improving work participation for adults with musculoskeletal conditions. Best Pract Res Clin Rheumatol 2012;26(5):733-42.

18. NHS Information. The clinical terms version 3 (The READ Codes). Birmingham: NHS Information Authority, 2000.

For peer review only - http://bmjopen.bmj.com/site/about/guidelines.xhtml

19. Ware J Jr, Kosinski M, Keller SD. A 12-item Short-Form Health Survey: construction of scales and preliminary tests of reliability and validity. Med Care 1996;34:220-233.

20. Lacey RJ, Lewis M, Jordan K, et al. Interrater reliability of scoring of pain drawings in a self-report health survey. *Spine* 2005;30(16):E455-8.

21. Wolfe F, Smythe HA, Yunus MB, et al. The American College of Rheumatology 1990 Criteria for the Classification of Fibromyalgia. Report of the Multicenter Criteria Committee. *Arthritis Rheum* 1990;33(2):160-72.

22. Kadam UT, Croft PR; North Staffordshire GP Consortium Group. Clinical multimorbidity and physical function in older adults: a record and health status linkage study in general practice. Fam Pract 2007;24:412-9.

23. Zigmond AS, Snaith RP. The hospital anxiety and depression scale. Acta Psychiatr Scand 1983;67:361-70.

24. Bergner M, Bobbitt RA, Carter WB, et al. The Sickness Impact Profile: development and final revision of a health status measure. *Med Care* 1981;19(8):787-805.

25. Hosmer DW, Lemeshow S. Applied Logistic Regression (2nd Edition). New York, NY: John Wiley & Sons; 2000.

26. Allaire S, Wolfe F, Niu J, et al. Work disability and its economic effect on 55-64-year-old adults with rheumatoid arthritis. Arthritis Rheum 2005;53(4):603-8.

27. Yelin EH, Henke CJ, Epstein WV: Work disability among persons with musculoskeletal conditions. Arthritis Rheum 29: 1322-1333, 1986

28. Allaire SH, Anderson JJ, Meenan RF. Reducing work disability associated with rheumatoid arthritis: identification of additional risk factors and persons likely to benefit from intervention. Arthritis Care Res 1996;9(5):349-57.

For peer review only - http://bmjopen.bmj.com/site/about/guidelines.xhtml

29. Lacaille D, Sheps S, Spinelli JJ, et al. Identification of modifiable work-related factors that influence the risk of work disability in rheumatoid arthritis. Arthritis Rheum 2004;51:843–52.

30. Reisine S, McQuillan J, Fifield J. Predictors of work disability in rheumatoid arthritis patients. A five-year followup. Arthritis Rheum 1995;38(11):1630-7.

31. Dunstan DA, Maceachen E. A Theoretical Model of Co-worker Responses to Work Reintegration Processes. J Occup Rehabil. 2013 Aug 3.

32 Gignac MA, Sutton D, Badley EM. Arthritis symptoms, the work environment, and the future: measuring perceived job strain among employed persons with arthritis. Arthritis Rheum. 2007;57(5):738-47.

33. Lacaille D, White MA, Backman CL, et al. Problems faced at work due to inflammatory arthritis: new insights gained from understanding patients' perspective. Arthritis Rheum 2007;57(7):1269-79

34. Sokka T, Pincus T. Markers for work disability in rheumatoid arthritis. The Journal of Rheumatology 2001;28(7):1718–22.

35. Løvvik C, Shaw W, Overland S, et al. Expectations and illness perceptions as predictors of benefit recipiency among workers with common mental disorders: secondary analysis from a randomised controlled trial. BMJ Open. 2014 Mar 3;4(3):e004321

36. Wilkie R, Blagojevic-Bucknall M, Jordan KP, et al. Onset of work restriction in employed adults with lower limb joint pain: individual factors and area-level socioeconomic conditions. J Occup Rehabil 2013 Jun;23(2):180-8.

37. Helmick CG, Felson DT, Lawrence RC, et al. Estimates of the prevalence of arthritis and other rheumatic conditions in the United States. Arthritis and Rheumatism 2008;58(1):15–25

For peer review only - http://bmjopen.bmj.com/site/about/guidelines.xhtml

38. Allaire SH, Li W, LaValley MP. Reduction of job loss in persons with rheumatic diseases receiving vocational rehabilitation: a randomized controlled trial. Arthritis Rheum 2003;48(11):3212-8.

39. Franche RL, Cullen K, Clarke J et al. Workplace-based return-to-work interventions: a stematic review of the quantitative literature. J Occup Rehabil 2005;15:607–631.

40. Gignac MA. Arthritis and employment: an examination of behavioral coping efforts to manage workplace activity limitations. Arthritis Rheum 2005;53(3):328-36.

41. Kievit AJ, Kuijer PPFM, Kievit RA, et al. Valid and Responsive Questionnaire to Score the Impact of Knee Complaints on Work Following Total Knee Arthroplasty: The WORQ. J ARTHROPLASTY 2014;ahead of print [PubMed]

42. Kievit AJ, van Geenen RCI, Kuijer PPFM, et al. A Cross-Sectional Multicenter Survey. J ARTHROPLASTY 2014;ahead of print [PubMed]

43. Gignac MA, Jetha A, Bowring J, et al. Management of work disability in rheumatic conditions: a review of non-pharmacological interventions. Best Pract Res Clin Rheumatol. 2012;26(3):369-86.

44. Coole C, Watson PJ, Drummond A. Staying at work with back pain: patients' experiences of work-related help received from GPs and other clinicians. A qualitative study. BMC Musculoskelet Disord 2010;11:190.

For peer review only

For peer review only - http://bmjopen.bmj.com/site/about/guidelines.xhtml

Table 1: Outcome and independent variables included in the analysis

	Time point when data was collected		
	Baseline	3-year follow-up	6- year follow-up
**Outcome**			
Expected work limitation			√
**Independent factors**			
**Health factors**			
Physical function		√	
Extent of pain	√	√	√
Comorbidity	√	√	√
Smoking	√		
Depression	√	√	√
Anxiety	√	√	√
Body mass Index	√	√	√
Cognitive impairment	√	√	√
Control of health	√		
**Socio-demographic**			
Age	√		
Gender	√		
Educational attainment	√		
Occupational classification	√		
Adequacy of income	√		
Live alone	√		
**Workplace factors**			
Work type			√
Work status			√
Physically demands of job			√
Flexible working			√
Use of aids and appliances			√
Opportunities to retrain			√
Work satisfaction			√

Table 2. Subject characteristics overall and by prediction status (n=297)

	All (n=297)	Will work to 69 without limitation (n=51)	Don't know if joint pain will limit (n =167)	Joint pain will limited or stop me working until 69 (n=79)
Age				
Median (Standard deviation)	54 (2.34)	57 (2.47)	54 (2.35)	53 (2.00)
Gender				
Male	134 (45.1)	32 (60.0)	64 (38.3)	38 (48.1)
Female	163 (54.9)	19 (40.0)	103 (61.7)	41 (51.9)
Educational attainment				
Further	63 (21.5)	11 (21.6)	39 (23.8)	13 (16.7)
School only	230 (78.5)	40 (78.4)	125 (76.2)	65 (83.3)
Live alone				
No	37 (12.9)	6 (12.2)	14 (8.6)	17 (22.7)
Yes	250 (87.1)	43 (87.8)	149 (91.4)	58 (77.3)
Occupational classification				
Managerial/Professional	73 (24.6)	17 (33.3)	40 (24.1)	16 (20.3)
Intermediate	57 (19.2)	10 (19.6)	28 (16.9)	19 (24.1)
Routine	166 (55.9)	24 (47.1)	98 (59.0)	44 (55.7)
Adequacy of income				
Adequate	129 (43.4)	22 (43.1)	85 (50.9)	22 (27.9)
Inadequate	168 (56.6)	29 (56.9)	82 (49.1)	57 (72.2)
Physical Function				
Median (Standard error)	85 (10.5)	90 (7.91)	85 (9.27)	65 (12.2)
Pain status				
None	15 (5.1)	2 (3.9)	12 (7.2)	1 (1.3)

For peer review only

Some	143 (48.2)	30 (58.8)	88 (52.7)	25 (31.7)
Widespread	139 (46.8)	19 (37.3)	67 (40.1)	53 (67.1)
Comorbidity				
Low comorbidity (0-3)	122 (41.1)	20 (39.2)	78 (46.7)	24 (30.4)
Multimorbidity (4 or more)	175 (58.9)	31 (60.8)	89 (53.3)	55 (69.6)
Depression				
Non-case (0-7)	228 (76.8)	44 (86.3)	138 (82.6)	46 (58.2)
Possible/probable case (8-21)	69 (23.2)	7 (13.7)	29 (17.4)	33 (41)
Anxiety				
Non-case (0-7)	121 (40.7)	27 (52.9)	65 (38.9)	29 (36.7)
Possible/probable case (8-21)	176 (59.3)	24 (47.1)	102 (61.1)	50 (63.3)
Body mass Index				
Normal (20-24.9 kg m^2)	68 (24.7)	13 (27.7)	42 (26.4)	13 (18.8)
Underweight (<20)	9 (3.3)	1 (2.1)	6 (3.8)	2 (2.9)
Overweight (25-29.9 kg	102 (37.1)	21 (44.7)	52 (32.7)	29 (42.0)
m^2)	96 (34.9)	12 (25.5)	59 (37.1)	25 (36.2)
Obese (>30 kg m^2)				
Cognitive complaint				
No cognitive complaint	122 (41.1)	28 (54.9)	75 (44.9)	19 (24.1)
Cognitive complaint	175 (58.9)	23 (45.1)	92 (55.1)	60 (76.0)
Smoking				
Never	119 (40.2)	22 (44.0)	73 (43.7)	24 (30.4)
Previous	119 (40.2)	20 (40.0)	68 (40.7)	31 (39.2)
Current	58 (19.6)	8 (16.0)	26 (15.6)	24 (30.4)
Control*				
Can control health	277 (93.9)	48 (94.1)	157 (95.2)	72 (91.1)
Can't control health	18 (6.1)	3 (5.9)	8 (4.9)	7 (8.9)
Work satisfaction				
Satisfied	198 (74.2)	43 (86.0)	115 (75.2)	40 (62.5)
Dissatisfied	69 (25.8)	7 (14.0)	38 (24.8)	24 (37.5)
Work type*				
Sedentary	88 (34.1)	18 (36.7)	53 (36.1)	17 (27.4)
Standing	71 (27.5)	9 (23.1)	41 (27.9)	21 (33.9)
Physical	81 (31.4)	20 (38.9)	45 (30.6)	16 (25.8)
Heavy manual	18 (7.0)	2 (4.6)	8 (5.4)	8 (12.9)
Physically demands of job				
Not physically demanding	171 (57.6)	36 (70.6)	101 (60.5)	34 (43.0)
Physically demanding	126 (42.4)	15 (29.4)	66 (39.5)	45 (57.0)
Flexible working				
Flexible	82 (27.6)	19 (37.3)	39 (23.4)	24 (30.4)
Not flexible	215 (72.4)	32 (62.7)	128 (76.6)	55 (69.6)
Use of aids and appliances				
Yes	151 (50.8)	32 (62.7)	79 (47.3)	40 (50.6)
No	146 (49.2)	19 (37.3)	88 (52.7)	39 (49.4)
Opportunities to retrain				
Yes	205 (69.0)	42 (82.4)	115 (68.9)	48 (60.8)
No	92 (31.0)	9 (17.6)	52(31.1)	31 (39.2)
Co-worker support				
Good co-worker support	277 (93.3)	51 (100)	154 (92.2)	72 (91.1)
Low co-worker support	20 (6.7)	0 (0)	13 (7.8)	7 (8.9)
Work amount				
Full-time	138 (46.5)	31 (60.8)	70 (41.9)	37 (46.8)
Part-time	121 (40.7)	15 (29.4)	81 (48.5)	25 (31.7)
Temp. work absence	38 (12.8)	5 (9.8)	16 (9.6)	15 21.5)

*Missing data

For peer review only - http://bmjopen.bmj.com/site/about/guidelines.xhtml

Table 3. Associations between health, demographic, socio-economic and workplace factors and significant future work limitation in primary care consulters for osteoarthritis; comparing EWL with no EWL odds ratios with 95% confidence intervals

			Multivariate model within each domain		Multivariate model including all domains	
	OR*	95%CI†	OR	95%CI	OR	95%CI
Health factors						
Physical function	**0.93**	**0.91, 0.96**	**0.94**	**0.91, 0.97**	**0.95**	**0.92, 0.97**
Extent of pain						
No pain	1		1		-	
Some	1.67	0.14, 19.5	0.44	0.03, 6.76	-	
Widespread	5.58	0.48, 65.1	0.84	0.05, 13.74	-	
Comorbidity						
Low comorbidity (0-3)	1		1		-	
Multimorbidity (4 or more)	1.48	0.71, 3.10	0.97	0.37, 2.56	-	
Smoking						
Never	1		1		1	
Previously	1.42	0.63, 3.18	2.19	0.73, 6.53,	1.52	0.51, 4.51
Currently	**2.75**	**1.02, 7.38**	2.99	0.79, 11.30	2.02	0.55, 7.40
Depression						
Non-case (0-7)	1		1		-	
Possible/probable case (8-21)	**4.51**	**1.81, 11.3**	1.27	0.35, 4.63	-	
Anxiety						
Non-case (0-7)	1		1		-	
Possible/probable case (8-21)	1.94	0.95, 3.97	1.08	0.37, 3.19	-	
Body mass Index						
Normal (20-24.9 kg m^2)	1		1		-	
Underweight (<20)	1.80	0.14, 23.4	1.15	0.02, 64.92	-	
Overweight (25-29.9 kg m^2)	1.13	0.39, 3.21	1.29	0.33, 5.09	-	
Obese (>30 kg m^2)	2.08	0.71, 6.10	1.33	0.31, 5.66	-	
Cognitive impairment						
No cognitive complaint	1		1		-	
Cognitive complaint	**3.84**	**1.81, 8.18**	1.98	0.70, 5.58	-	
Control of health						
Can control health	1		1		-	
Can't control health	1.56	0.38, 6.31	1.64	0.29, 9.36	-	
R^2			0.311			
C- statistic			0.8641			
Socio-demographic						
Age	0.69	0.58, 0.82	0.67	0.56, 0.81	**0.74**	**0.60, 0.91**
Gender						
Male	1		1		-	
Female	1.82	0.89, 3.72	1.37	0.58, 3.22	-	
Educational attainment						
Further	1		1		-	
School only	1.38	0.56, 3.36	1.12	0.34, 3.64	-	
Occupational classification						
Managerial/Professional	1		1		-	
Intermediate	2.01	0.72, 5.63	2.27	0.64, 8.12	-	
Routine	1.94	0.84, 4.53	2.26	0.74, 6.90	-	
Adequacy of income						
Adequate	1		1		-	
Inadequate	1.97	0.94, 4.12	2.01	0.84, 4.81	-	
Live alone						
No	1		1		-	
Yes	0.48	0.17, 1.31	0.50	0.16, 1.56	-	
R^2			0.175			
C-statistic			0.7636			
Workplace factors						
Work type						
Sedentary	1		1		-	
Standing	2.47	0.89, 6.88	2.32	0.60, 7.92	-	
Physical	0.85	0.33, 2.15	0.46	0.13, 1.59	-	
Heavy manual	4.24	0.79, 22.8	2.38	0.30, 18.7	-	
Work status						
Full-time	1		1		-	
Part-time	1.40	0.63, 3.10	1.07	0.40, 2.87	-	

For peer review only

For peer review only - http://bmjopen.bmj.com/site/about/guidelines.xhtml

Off work	2.85	0.94, 8.60	0.26	0.04, 1.91	-	
Physically demands of job						
Not physically demanding	1		1		-	
Physically demanding	3.18	1.50, 6.72	1.81	0.63, 5.21	-	
Flexible working						
Flexible	1		1		-	
Not flexible	1.36	0.65, 2.86	1.77	0.62, 5.04	-	
Use of aids and appliances						
Yes	1		1		-	
No	1.64	0.80, 3.37	1.06	0.43, 2.65	-	
Opportunities to retrain						
Yes	1		1		-	
No	3.01	1.29, 7.05	1.99	0.72, 5.45	-	
Work satisfaction						
Satisfied	1		1		1	
Dissatisfied	3.69	1.43, 9.49	4.78	1.57, 14.6	2.08	0.66, 6.51
R^2			0.116		0.296	
C-statistic			0.7785		0.8418	

*Odds ratio; † 95 percent confidence interval;

FIGURE LEGENDS

Figure 1. Flow diagram of participants for the longitudinal analysis

For peer review only

Anticipated significant work limitation in primary care consulters with osteoarthritis: a prospective cohort study

Ross Wilkie[1], Chris Phillipson[2], Elaine M Hay[1], Glenn Pransky[3]

[1]Arthritis Research UK Primary Care Centre, Primary Care Sciences, Keele University, Keele, Staffordshire, United Kingdom, ST5 5BG.
[2] School of Social Sciences, The University of Manchester, Manchester, United Kingdom, M13 9PL
[3] Center for Disability Research, Liberty Mutual Research Institute, 71 Frankland Rd., Hopkinton MA 01748. United States.

Address for reprint requests and for corresponding author
Dr. Ross Wilkie
Arthritis Research UK Primary Care Centre, Primary Care Sciences, Keele University, Keele, Staffordshire, United Kingdom, ST5 5BG.
Email: r.wilkie@keele.ac.uk
Tel: +44 1782 734845
Fax: +44 1782 583911

Short title: Expected work loss in osteoarthritis consulters

Key indexing terms: osteoarthritis, work disability, epidemiology, primary health care, joint pain

Conflicts of interest: The authors declare no conflicts of interest

Funding: The NorStOP study was funded by a programme Grant from the MRC UK (grant code G9900220)

For peer review only

For peer review only - http://bmjopen.bmj.com/site/about/guidelines.xhtml

Abstract

Objective: To describe the prevalence of expected work limitations (EWL) prior to future retirement age in osteoarthritis consulters, and the associated health, socio-demographic and workplace factors.

Design: Population-based prospective cohort study.

Setting: General practices in Staffordshire, England.

Participants: 297 working adults aged 50 to 65, who had consulted primary care for osteoarthritis.

Outcome: EWL was defined using a single question, "Do you think joint pain will limit your ability to work before you reach 69 years old".

Results: 51 (17.2%) indicated that joint pain would not limit their ability to work until 69, 79 (26.6%) indicated EWL and 167 (56.2%) did not know if joint pain would limit work before 69. In bivariate analysis, physical function (Odds ratio 0.93; 95% confidence interval 0.91, 0.96), depression (4.51; 1.81, 11.3), cognitive complaint (3.84; 1.81, 8.18), current smoker (2.75; (1.02, 7.38), age (0.69; 0.58, 0.82), physically-demanding job (3.18; 1.50, 6.72), no opportunities to retrain (3.01; 1.29, 7.05) and work dissatisfaction (3.69; 1.43, 9.49) were associated with EWL. The final multivariate model, included physical function and age.

Conclusions: Only one in five osteoarthritis consulters expected that joint pain would not limit their work participation before 69 years old. Given the expectation for people to work until they're older, the results highlight the increasing need for clinicians to include work participation in their consultation and implement strategies to address work loss/limitation. Targeting pain related functional limitation and effective communication with employers to manage workplace issues could reduce EWL.

For peer review only

Article summary

- Osteoarthritis is a common reason for primary care consultation and joint pain is strongly associated with ageing and disability. Loss of work participation is one form of disability that will become more important as adults work to older ages, due to rising state pension age, and have greater financial needs resulting from inadequate retirement resources.
- The aim of this study was to estimate the proportion of working age adults with osteoarthritis who predict that joint pain would limit their work or stop them working prior to a possible future pension age of 69 (i.e. EWL) and identify health, socio-demographic and workplace factors associated with EWL.

Key messages

- Only one in five consulters for osteoarthritis expected that joint pain would not limit their work participation before 69 years old.
- Low physical function, being a current smoker, depression, and several workplace factors - physically-demanding job, work dissatisfaction, and poor co-worker support – were associated with anticipated early work limitation.

Strengths and Limitations

- The sample is representative of primary care consulters with physician diagnosed osteoarthritis.
- The outcome is based on individual's expectations but this can be highly predictive of future work loss/limitation and drive consultation for health care.

For peer review only - http://bmjopen.bmj.com/site/about/guidelines.xhtml

Introduction

Osteoarthritis is the most common joint condition in adults and globally is the fastest increasing major heath condition [1]. It is a common reason for primary care consultation (one in twenty consultations in adults aged between 45 and 65 per year is for osteoarthritis [2]) and is recognised as one of the leading and rapidly growing causes of disability [3]. Its most disabling manifestation (joint pain) is strongly associated with ageing [4] and with the commonest forms of disability [5-9].

Work restriction is one form of disability that will become more important for those with osteoarthritis and joint pain because increases in state pension age in many developed countries means that most adults can expect a need to continue working at older ages than before [10]. Normal retirement age in North America and Europe has increased, and is expected to rise further to 69 and beyond [11]. However the extent to which participation in work will be limited by health-related problems, resulting in significant work limitation in terms of absenteeism and presenteeism (remaining in work but with limitation and reduced productivity) is unclear [12]. The increasing prevalence of chronic health conditions, especially osteoarthritis, in persons near to retirement age raises questions about viability of attempts to extend working life. Several studies of expectations of future work loss are predictive of future work outcomes [13,14]. Identifying the prevalence and predictors of expected work limitations (EWL) in this group of patients, particularly those that are amenable to change, will inform management and possible preventative strategies for future work limitation. The aim of this study was to estimate the proportion of working age adults with osteoarthritis who predict that joint pain would limit their work or stop them working (i.e. EWL) prior to a possible future pension age of 69. In addition health, socio-demographic

For peer review only - http://bmjopen.bmj.com/site/about/guidelines.xhtml

or workplace factors associated with EWL, especially those amenable to change, were explored, to identify potential targets to manage and prevent EWL [15-17].

Method

Study population

The North Staffordshire Osteoarthritis project (NorStOP) is a population-based prospective cohort study. The NorStOP sampling frame comprised all individuals aged 50 years and over who were registered to receive care from one of six general practices in North Staffordshire, England, United Kingdom (UK). In 2002, adults aged who gave their written consent for medical record review were followed up over 6 years for consultation to primary care. They were also mailed questionnaires at three and six years; reminders were sent at two and four weeks after the initial mailing. The North Staffordshire Local Research Ethics Committee approved this study.

Analyses for this paper included those who (i) consulted for osteoarthritis during the study period (starting 18 months before the baseline questionnaire was administered, and continuing through the time of the final follow-up questionnaire (i.e. from 2000 to 2008)), (ii) were of working age (less than 65 years old) and in employment at the six year follow-up and (iii) completed the item on expected work limitation prior to 69 years old (EWL) at six year follow-up. Over the study period there were 923 adults who had consulted for osteoarthritis and were of working age at 6 year follow-up. Of this group 398 had retired before state retirement age, 13 were unemployed, 31 were homemakers, leaving 481 who were in employment and thus eligible for the study. Of these, 184 did not complete the item on future work limitation, leaving complete data for 297 participants (adjusted response 61.7%) (Figure 1). Compared to those subjects who had consulted for osteoarthritis but did not complete the item on future work limitation questionnaire (n=184) those included in the analysis (n=297)

For peer review only - http://bmjopen.bmj.com/site/about/guidelines.xhtml

were more likely to be female (p=0.02) and have an adequate income p=0.052) but no more likely to be older (p=0.19), have better physical (p=0.91) or mental health (p=0.21), or have gone onto further education (p=0.52) or have an adequate income (p=0.052).

Identification of osteoarthritis

General practitioners in the study used the ReadEAD system to code all reasons for clinical encounters in primary care consultations [18]. The Read codes cross-map to ICD9/ICD-10 (for diseases). Morbidity data (i.e. symptoms and diseases) in this system are grouped under 19 main ReadEAD chapters. Data collected at the second hierarchical level or above was used to identify diagnostic groups, and these were aggregated starting 18 months before the baseline questionnaire was administered, and continuing through the time of the final follow-up questionnaire. Individuals were defined as having osteoarthritis if they had at least one consultation during this period primarily for osteoarthritis based on Read codes (N05 category) for primary care consultations [18]. As osteoarthritis is a long-standing, gradually progressive chronic condition, it was assumed that a clinician-established diagnosis at some point during the study period implied that OA was likely present at least to some degree during the entire period of observation.

Outcome measure

Expected work limitation (EWL) was defined using a single question at six-year follow-up, "do you think joint pain will limit your ability to work before you reach 69 years old" (will limit or stop me/ don't know/ won't limit).

Independent factors

For peer review only

Health factors were measured across the six year study period, socio-demographic factors at three year follow-up and workplace factors, were measured retrospectively at six year follow-up (Table 1).

Health Factors

Physical function was measured at each time point using the physical functioning scale of the Medical Outcomes Study Short Form-36; score range: 0-100, higher scores indicating better function [19]. Items measured the limitation in the individual's capacity to complete basic tasks such as lifting and walking. Scores at each time point were highly correlated (i.e. between baseline and three year follow-up r=0.71; between three and six year follow-up r=0.75). Physical function score at three year follow-up (the middle point of data collection) was used in this analysis. The extent of musculoskeletal pain was measured by responders shading painful areas (0-44) on a full body diagram (front and back views). These methods to determine the location and extent of pain are commonly used in population based studies of pain, and have been shown to be valid and reliable [20]. Using these pain drawings, participants were classified into one of three groups (none, some and widespread). The widespread group were those participants that satisfied the criteria for widespread pain included in the American College of Rheumatology 1990 criteria for fibromyalgia [21] at baseline, three or six years. These criteria require pain to be present above and below the waist, in the right and left hand sides of the body and in the axial skeleton; remaining participants who reported pain at any time point that did not satisfy the criteria for widespread were classified as having "some pain" and those participants who did not report pain at all were classified as "no pain".

For peer review only - http://bmjopen.bmj.com/site/about/guidelines.xhtml

Comorbidity was identified using Read diagnostic codes from primary care consultations. Multimorbidity was defined as 4 or more comorbidities (different major diagnostic groups) in the 2 years prior to baseline, between baseline and 3 year follow-up or between three and 6 year follow-up [22]. Anxiety and depression during the previous week were measured using the Hospital Anxiety and Depression Scale (HADS) - raw scores categorised individuals as non-cases (0-7) or possible/probable cases (8-21); depression was defined as possible or probable case at any of the three time points [23]. Self-reported height and weight wasere categorised into standard BMI groups (i) normal weight (BMI 20-24.9kgm^{-2}), (ii) underweight (BMI <20kgm^{-2}), (iii) overweight (BMI 25 – 29.9 kgm^{-2}) and (iv) obese (BMI ≥30kgm^{-2}). Cognitive complaint was measured using the Alertness Behaviour Subscale of the Sickness Impact Profile [24]. This scale has 10 items that ask about alertness and ability to concentrate. Each item was scored as 0 (no cognitive complaint) or 1 (cognitive complaint) with raw additive scores categorised to indicate "no cognitive complaint" (score of 0) and "cognitive complaint" (score >0); individuals were identified as having cognitive complaint if they had a score >0 at any of the three time points. Perceived control of health was measured using a single item at baseline (There is a lot I can do to control my health yes/no). Participants were also asked to report their smoking status at baseline (current, previous, never).

Socio-demographic Factors

Demographic and socio-economic details included age, gender, educational attainment (those who finished their education on leaving school; those who went onto further education such as college or university), occupational class (managerial or professional (chief executive, professor), intermediate (e.g. paramedics, technicians), routine (machine operator, childcare worker), adequacy of income (Thinking about the cost of living as it affects you, which of

For peer review only - http://bmjopen.bmj.com/site/about/guidelines.xhtml

these descriptions best describes your situation; adequate/inadequate) and living status (live alone/live with others).

Workplace Factors

Single items on the 6-year follow-up questionnaire measured workplace characteristics. Items included work type (Which of the following statements best describes the work that you do in your current job; sedentary occupation/standing occupation/physical work/heavy manual work), work status (current employment status: full-time/part-time/temporarily off work (e.g. due to sickness), physically demanding employment (Thinking over the last 30 days: Is your work physically demanding; not physically demanding/physically demanding), flexible working (My hours of work are flexible; flexible/not flexible), co-worker support (My work colleagues are supportive: good/low), work satisfaction (How satisfied are you with your current job satisfaction/dissatisfied), opportunities to retrain (There are opportunities to retrain and develop my skills; yes/no) and able to use aids and appliances and adapt the workplace (I can use aids and appliances to help me do my job or adapt my work; yes/no).

Statistical analysis

First, the distribution of health, socio-demographic and workplace factors was examined by prediction of the ability to work until 69 with differences tested for significance using Chi-square or Kruskall Wallis tests where appropriate. Regression analyses then focused on identifying factors associated with significant future work loss (will be limited in ability to work, or will be unable to work until age 69 because of joint pain (i.e. EWL)); compared to no expected work loss. Bivariate and multivariate logistic regression models were constructed to examine the relationship between each health, socio-demographic and workplace factor and EWL. Following bivariate analysis, multivariate models included all of the factors within each category (i.e. all health factors in one model, all socio-demographic

For peer review only - http://bmjopen.bmj.com/site/about/guidelines.xhtml

in the second model and all of the workplace factors in the third model for each outcome). Factors independently associated with EWL within each category were then included in a final multivariate model. To evaluate model fit, concordance indexes (C-statistic) were calculated for each model. A C-statistic of 0.50 indicates the predictive ability of a model to be no better than chance, 0.7 indicates reasonable, 0.8 indicates high and 1.0 indicates perfect predictive ability [25].

Stata version 11 was used for all analyses. The results of the analyses are presented as odds ratios (OR) with 95% confidence intervals (CI). For the regression analyses the "won't be limited" group was classified as the referent category.

Results

Of the 297 consulters for osteoarthritis included in the analysis, 51 (17.2%) indicated that joint pain would not limit their ability to work until 69 years, 79 (26.6%) indicated that joint pain would limit or stop them working before 69 years (EWL) and 167 (56.2%) indicated that they did not know if they would have EWL before 69 years. Those who indicated EWL (median age 53 years) were younger than those who did not know (median age 54 years) or did not predict EWL (median age 57; p=0.0001) (Table 21). Women were more likely to indicate that they did not know or would have EWL (p=0.01). There was no significant difference among the three groups for educational attainment (p=0.44) or occupational classification (p=0.10). Notably all responders with low co-worker support predicted developing EWL before age 69 years.

Table 32 shows the results of the regression analysis comparing anticipated EWL to no EWL. In the bivariate analysis, health, socio-economic and workplace factors were associated with

For peer review only - http://bmjopen.bmj.com/site/about/guidelines.xhtml

EWL before age 69 years. Of the health factors, depression (odds ratio 4.51; 95% confidence interval 1.81, 11.3), cognitive complaint (3.84; 1.81, 8.18) and being a current smoker (2.752; (1.02, 7.38) were associated with EWL onset prior to 69 years. Increasing physical function (0.93; 0.91, 0.96) was protective against EWL onset prior to 69 years. In the multivariate analysis of health factors only increasing physical function (0.94; 0.90, 0.97) was associated with EWL onset before 69 years. Of the socio-economic factors, only age (0.69; 0.58, 0.82) was associated with EWL. Of the workplace factors, a physical demanding job (3.18; 1.50, 6.72), no opportunities to retrain (3.01; 1.29, 7.05) and work dissatisfaction (3.69; 1.43, 9.49) were associated with EWL. In the multivariate analysis of workplace factors, a physically demanding job and work satisfaction were independently associated with EWL. In the final multivariate model, combining significant factors from the health, socio-economic and workplace factors, the association with work dissatisfaction attenuated to insignificance (adjusted odds ratio 2.08; 95% confidence interval 0.66, 6.51). Physical function (0.95; 0.92, 0.97) and age (0.74; 0.60, 0.91) remained independently associated with EWL; for every 1 point increase in physical function the odds of EWL increased by 7% and for every 1 year increase in age, the likelihood of EWL decreased by 45%. The model fit of the final model was 0.8418.

For peer review only

Discussion

Principal findings

This study is the first to examine the factors associated with an expectation of having significant future work limitation (EWL) for employed patients who consult primary care physicians for osteoarthritis. Physical function, being a current smoker, depression, and several workplace factors - physically-demanding job, work dissatisfaction, and poor co-

For peer review only - http://bmjopen.bmj.com/site/about/guidelines.xhtml

worker support – were associated with anticipated EWL. Only one in five osteoarthritis consulters (17.2%) indicated that joint pain would not limit their ability to work until age 69. Given the high and increasing prevalence of osteoarthritis, the number of consultations for this disorder, and the anticipated extension of working lives due to changes in retirement policy, these results raise significant concerns, while suggesting potential areas for intervention.

The proportion of osteoarthritis cases who expected work limitation was higher compared to previous studies. Using a national US sample of all workers, Theis and Murphy found that only a third of those with physician-diagnosed arthritis reported work limitations [15]. Unlike the current study, their sample was not restricted to employed persons, and did not ask about expected ability to work in the future. Studies of rheumatoid arthritis patients have found that premature work loss in persons in a similar age group is common, affecting up to 90% of persons with this diagnosis, and leads to significant economic and social consequences [26]. Our finding of a similar high rate of expected work loss in persons with OA suggests that the societal impact in this much larger group will be significant as well.

Physical function was highly protective against EWL. Reduced physical function has been found to be , similar to findings observed in other studieassociated with s of current work limitations in persons with any type of arthritis [15], musculoskeletal disorders in general [27] or those with a specific arthritis diagnosis [28,29]. This indicates an important mismatch between individual capabilities and work demands, that is more important than pain by itself. High work physical demands have long been recognized as an important risk factor for subsequent work disability in rheumatoid arthritis [27]. Some have suggested that control over work demands might be more important than the absolute level of work demands, but

For peer review only - http://bmjopen.bmj.com/site/about/guidelines.xhtml

these studies did not evaluate the level of job physical demands [30]. Work dissatisfaction was also significant, although this problem was reported in relatively few respondents. There may be several dimensions of work dissatisfaction – not only being dissatisfied at work, but also a preference to be at home instead [30]. Similarly, lack of co-worker support was a problem in only a relatively small number of persons, but all who reported low co-worker support expected to have EWL. All of those who reported low co-worker support also indicated at least one significant health problem (i.e. widespread pain, multimorbidity, depression or anxiety). The importance of co-worker support in those returning to work after injury or illness, across a range of conditions, has recently been recognized [31], and low co-worker support has been associated with greater job strain and work loss in workers with arthritis [32,33]. This may not be about Alternatively, this finding may be less related to co-worker support but be a description than the particular nature of worktheir job, such as people working on their own, or in job types in which cannot that have little involvement involve with others. If working trends in the arrangements of jobspractices in the future means that more older adults with osteoarthritis are working on their ownthis changes in the future the significance of low co-worker support implications of this will increase.

We did not see the effects of low education (e.g. [15]) or comorbidities reported by others. Education may not have a large impact compared to actual job physical demands. Flexible work was not a factor, although it has been identified in other studies as a significant predictor of staying at work with RA [343]. This difference may be due to the less specific nature of the question in this study, compared with other investigations.

For peer review only - http://bmjopen.bmj.com/site/about/guidelines.xhtml

The majority of participants (52.6%) could not predict whether pain would limit or stop their ability to work until the new retirement age. In additional analysis, a forward stepwise logistic regression model of health, socio-demographic and workplace factors was constructed to identify which factors were associated with being unable to predict whether pain would limit the ability to work.. Lower age, being in part time work and being unable to use aids and appliances were the factors significantly associated with being unable to predict. Their responses may be due to some uncertainty about the future progression of their condition, or about whether or not employment until age 69 would be required. Notably the greater likelihood for women to indicate "don't know" was explained by these factors. The role of aids and appliances again indicates the importance of the workplace to allow individuals to self-manage their pain to optimise performance.

Strengths and weaknesses

The strength of this study's longitudinal design enables prospective identification of factors associated with EWL in a clinically relevant primary care population. The sample is representative of primary care consulters with physician diagnosed osteoarthritis, relevant to primary care practices. Other studies have been limited to patients from rheumatology practices or rehabilitation clinics, a less representative sample of osteoarthritis patients.

There are limitations to this study. A range of factors have been identified as potentially linked to future employment loss in osteoarthritis, and some were not included in the information collected for this study, such as extent of joint involvement, success of current coping strategies, opportunities for part-time work or retirement, importance of work role, social support outside of the workplace, and extent of workplace accommodations specific to their osteoarthritic condition [16]. In addition, overall pain levels, illness perceptions and

For peer review only - http://bmjopen.bmj.com/site/about/guidelines.xhtml

health beliefs have not been included, which may have considerable impact on predicted work outcomes [35] We chose to measure extent of pain rather than pain level, as we have previously found extent of pain to be associated with poor work outcomes ~~there is some evidence that extent is a more stable measure,~~ and this may be more reflective of total arthritis impact on physical function than pain intensity [36]. However, clinical measures may not be as important in predicting work disability, compared to measure of function, psychosocial and workplace factors [344]. The C-statistics for the health (0.8641), socio-demographic (0.7636), workplace (0.7785) and final (0.8418) models indicate a reasonable or high ability to predict EWL. The factors included here are those thought to be most important in maintaining employment in chronic musculoskeletal conditions. Data on most variables was by self-report, but validated instruments were used to measure anxiety, depression and pain ~~interference~~extent. The outcome is based on individual's expectations but this can be highly predictive of future work loss/limitation and drive consultation for health care [15-17]. We did not have radiographic or detailed information on the extent of OA, but the intention of the study was to describe a typical, heterogeneous group of patients with OA as seen in primary care practice. Measurement of predictors at three time points may not reflect changes in these factors during follow-up. Workplace factors were measured only at six year follow-up, and may be out of sequence with health factors, but give a sense of current workplace status at the same time that the question on anticipated EWL was asked. As with any cohort study, non-completion of the items may affect estimates, however based on comparisons between those included in the analysis and those who did not complete the EWL item, such effects are likely to be small. The area covered by the study is more deprived on health, education, and employment, but with fewer barriers

For peer review only - http://bmjopen.bmj.com/site/about/guidelines.xhtml

to housing and services, than England as a whole, but again the potential effect of this on estimates will be small.

Clinical implications

With current disease prevalence and economic trends, and increasing prevalence of osteoarthritis, clinicians will undoubtedly have more consultations with persons concerned about their ability to stay at work [3~~5~~7]. Studies in rheumatoid arthritis suggest that a proactive approach – identification and intervention before work loss has occurred – can be effective in preventing subsequent work loss [3~~6~~8]. In this study, ~~the~~ health ~~and workplace~~ problems were present well in advance of when EWL was measured. For example, the high correlation between physical function scores at baseline and three years (0.71) indicates that low physical function was experienced six years before EWL was measured. This indicates that there is a lot of time to intervene with available strategies [3~~7~~9]. Certain types of coping strategies may be more effective than others in maintaining employment in persons with arthritis, especially anticipating challenges in the workplace and formulating strategies to deal with them in advance [40~~38~~]. Given the importance of osteoarthritis-related physical limitations at work as a predictor of EWL, a positive screening question for these limitations should lead to a more in-depth discussion about accommodations and employment options, and perhaps a referral to a vocational expert [41,42]. The negative association with inadequate co-worker support suggests that employer engagement in creating a supportive work environment will also be a major factor in work retention for these patients [33]. Addressing the needs of employed osteoarthritis patients within the context of a broader psychosocial model of disease ensures a broader and more relevant perspective on the causes and prevention opportunities for subsequent work loss [17].

For peer review only

For peer review only - http://bmjopen.bmj.com/site/about/guidelines.xhtml

There are still many unanswered questions about the optimal nature, timing and duration of interventions designed to maintain employment, and how to engage treating clinicians as positive contributors; thus further studies will be needed in order to understand how best to address these problems [43,44~~39, 40~~].

Conclusion

This observational study suggests ~~only one in~~ 4 out of 5 ~~five~~ consulters to primary care with osteoarthritis ~~do not~~ expect their joint pain to limit their work participation prior to future pension age, and that younger age and greater arthritis-related physical limitations are the main factors associated with this expected outcome. Given the expectation for people to work until they are older with osteoarthritis, the results highlight the increasing need for clinicians to include work participation in their consultation and implement strategies to prevent work limitation. Targeting pain related functional limitation and effective communication with employers to manage workplace issues could reduce the expectation of future work limitation.

ACKNOWLEDGEMENTS

We would like to thank the administrative and health informatics staff at Keele University's Arthritis Research UK Primary Care Centre and the doctors and staff of the participating general practices.

FINANCIAL DISCLOSURE: This study is supported financially by the Medical Research Council, UK (grant code: G9900220) and by the North Staffordshire Primary Care R&D Consortium.

DATA SHARING: The Arthritis Research UK Primary Care Research Centre has established data sharing arrangements to support joint publications and other research

For peer review only

For peer review only - http://bmjopen.bmj.com/site/about/guidelines.xhtml

collaborations. Applications for access to anonymized data from our research databases are reviewed by the Centre's Data Custodian and Academic Proposal (DCAP) Committee and a decision regarding access to the data is made subject to the NRES ethical approval first provided for the study and to new analysis being proposed. Further information on our data sharing procedures can be found on the Centre's website (http://www.keele.ac.uk/pchs/publications/datasharingresources/) or by emailing the Centre's data manager (data-sharing-pcs@cphc.keele.ac.uk).

FIGURE LEGENDS

Figure 1. Flow diagram of participants for the longitudinal analysis

References

1. Murray CJ, Vos T, Lozano R, Naghavi M, Flaxman AD, Michaud C et al. Disability-adjusted life years (DALYs) for 291 diseases and injuries in 21 regions, 1990-2010: a systematic analysis for the Global Burden of Disease Study 2010. Lancet 2012;380(9859):2197-223.

2. Jordan K, Clarke AM, Symmons DP, Fleming D, Porcheret M, Kadam UT, Croft P. Measuring disease prevalence: a comparison of musculoskeletal disease using four general practice consultation databases. Br J Gen Pract 2007;57:7-14.

3. Arden N, Nevitt MC. Osteoarthritis: epidemiology. Best Pract Res Clin Rheumatol 2006;20:3-25.

4. Doherty M. Risk factors for progression of knee osteoarthritis. Lancet 2001;358:775-6.

5. Jordan JM, Luta G, Renner JB, Linder GF, Dragomir A, Hochberg MC, Fryer JG. Self-reported functional status in osteoarthritis of the knee in a rural southern community: the role of sociodemographic factors, obesity, and knee pain. Arthritis Care Res 1996;9:273-8.

For peer review only - http://bmjopen.bmj.com/site/about/guidelines.xhtml

6. Jinks C, Jordan K, Croft P. Osteoarthritis as a public health problem: the impact of developing knee pain on physical function in adults living in the community: (KNEST 3). Rheumatology 2007;46(5):877-81.

7. Felson DT, Lawrence RC, Dieppe PA, Hirsch R, Helmick CG, Jordan JM, Kington RS, Lane NE, Nevitt MC, Zhang Y, Sowers M, McAlindon T, Spector TD, Poole AR, Yanovski SZ, Ateshian G, Sharma L, Buckwalter JA, Brandt KD, Fries JF. Osteoarthritis: new insights. Part 1: the disease and its risk factors. Ann Intern Med 2000;133:635-46.

8. Wilkie R, Peat G, Thomas E, Croft PR. Factors associated with restricted mobility outside the home in community-dwelling adults aged 50 years and over with knee pain – an example of use of the International Classification of Functioning to investigate participation restriction. Arthritis Rheum 2007;57(8):1381-9.

9. Odding E, Valkenburg HA, Stam HJ, Hofman A. Determinants of locomotor disability in people aged 55 years and over: The Rotterdam study. Eur J Epidemiol 2002;17:1033-41.

10. Helman R, Copeland C, VanDerhei J. The 2012 Retirement Confidence Survey: job insecurity, debt weigh on retirement confidence, savings. EBRI Issue Brief 2012;369:5-32,1.

11. Organisation for Economic Co-operation and Development. OECD Pension Outlook 2012. http://www.oecd.org/pensions/private-pensions/50560110.pdf (accessed August 2013).

12. Bieleman HJ, Bierma-Zeinstra SM, Oosterveld FG, Reneman MF, Verhagen AP, Groothoff JW. The effect of osteoarthritis of the hip or knee on work participation. J Rheumatol 2011;38(9):1835-43.

13. Fadyl J, McPherson K. Return to work after injury: a review of evidence regarding expectations and injury perceptions, and their influence on outcome. J Occup Rehabil. 2008;18(4):362-74.

For peer review only - http://bmjopen.bmj.com/site/about/guidelines.xhtml

14. Reme SE, Hagen EM, Eriksen HR. Expectations, perceptions, and physiotherapy predict prolonged sick leave in subacute low back pain. BMC Musculoskelet Disord 2009;10:139.

15. Theis KA, Murphy L, Hootman JM, Helmick CG, Yelin E. Prevalence and correlates of arthritis-attributable work limitation in the US population among persons ages 18-64: 2002 National Health Interview Survey Data. Arthritis Rheum 2007;57(3):355-63.

16. Hoving JL, van Zwieten MC, van der Meer M, Sluiter JK, Frings-Dresen MH. Work participation and arthritis: a systematic overview of challenges, adaptations and opportunities for interventions. Rheumatology 2013;52(7):1254-64

17. Wilkie R, Pransky G. Improving work participation for adults with musculoskeletal conditions. Best Pract Res Clin Rheumatol 2012;26(5):733-42.

18. NHS Information. The clinical terms version 3 (The READ Codes). Birmingham: NHS Information Authority, 2000.

19. Ware J Jr, Kosinski M, Keller SD. A 12-item Short-Form Health Survey: construction of scales and preliminary tests of reliability and validity. Med Care 1996;34:220-233.

20. Lacey RJ, Lewis M, Jordan K, Jinks C, Sim J. Interrater reliability of scoring of pain drawings in a self-report health survey. *Spine* 2005;30(16):E455-8.

21. Wolfe F, Smythe HA, Yunus MB, Bennett RM, Bombardier C, Goldenberg DL, et al. The American College of Rheumatology 1990 Criteria for the Classification of Fibromyalgia. Report of the Multicenter Criteria Committee. *Arthritis Rheum* 1990;33(2):160-72.

For peer review only

22. Kadam UT, Croft PR; North Staffordshire GP Consortium Group. Clinical multimorbidity and physical function in older adults: a record and health status linkage study in general practice. Fam Pract 2007;24:412-9.

23. Zigmond AS, Snaith RP. The hospital anxiety and depression scale. Acta Psychiatr Scand 1983;67:361-70.

24. Bergner M, Bobbitt RA, Carter WB, Gilson BS. The Sickness Impact Profile: development and final revision of a health status measure*. Med Care* 1981;19(8):787-805.

25. Hosmer DW, Lemeshow S. Applied Logistic Regression (2nd Edition). New York, NY: John Wiley & Sons; 2000.

26. Allaire S, Wolfe F, Niu J, Lavalley M, Michaud K. Work disability and its economic effect on 55-64-year-old adults with rheumatoid arthritis. Arthritis Rheum 2005;53(4):603-8.

27. Yelin EH, Henke CJ, Epstein WV: Work disability among persons~~Demons~~ with musculoskeletal conditions. Arthritis Rheum 29: 1322-1333, 1986

28. Allaire SH, Anderson JJ, Meenan RF. Reducing work disability associated with rheumatoid arthritis: identification of additional risk factors and persons likely to benefit from intervention. Arthritis Care Res 1996;9(5):349-57.

29. Lacaille D, Sheps S, Spinelli JJ, Chalmers A, Esdaile JM. Identification of modifiable work-related factors that influence the risk of work disability in rheumatoid arthritis. Arthritis Rheum 2004;51:843–52.

30. Reisine S, McQuillan J, Fifield J. Predictors of work disability in rheumatoid arthritis patients. A five-year followup. Arthritis Rheum 1995;38(11):1630-7.

31. Dunstan DA, Maceachen E. A Theoretical Model of Co-worker Responses to Work Reintegration Processes. J Occup Rehabil. 2013 Aug 3.

For peer review only

For peer review only - http://bmjopen.bmj.com/site/about/guidelines.xhtml

32 Gignac MA, Sutton D, Badley EM. Arthritis symptoms, the work environment, and the future: measuring perceived job strain among employed persons with arthritis. Arthritis Rheum. 2007;57(5):738-47.

33. Lacaille D, White MA, Backman CL, Gignac MA. Problems faced at work due to inflammatory arthritis: new insights gained from understanding patients' perspective. Arthritis Rheum 2007;57(7):1269-79

34. Sokka T, Pincus T. Markers for work disability in rheumatoid arthritis. The Journal of Rheumatology 2001;28(7):1718–22.

35. Løvvik C, Shaw W, Overland S, Reme SE. Expectations and illness perceptions as predictors of benefit recipiency among workers with common mental disorders: secondary analysis from a randomised controlled trial. BMJ Open. 2014 Mar 3;4(3):e004321

36. Wilkie R, Blagojevic-Bucknall M, Jordan KP, Pransky G. Onset of work restriction in employed adults with lower limb joint pain: individual factors and area-level socioeconomic conditions. J Occup Rehabil 2013 Jun;23(2):180-8.

37~~5~~. Helmick CG, Felson DT, Lawrence RC, Gabriel S, Hirsch R, Kwoh CK, et al. Estimates of the prevalence of arthritis and other rheumatic conditions in the United States. Arthritis and Rheumatism 2008;58(1):15–25

38~~6~~. Allaire SH, Li W, LaValley MP. Reduction of job loss in persons with rheumatic diseases receiving vocational rehabilitation: a randomized controlled trial. Arthritis Rheum 2003;48(11):3212-8.

39~~7~~. Franche RL, Cullen K, Clarke J et al. Workplace-based return-to-work interventions: a s~~ys~~tematic review of the quantitative literature. J Occup Rehabil 2005;15:607–631.

40~~38~~. Gignac MA. Arthritis and employment: an examination of behavioral coping efforts to manage workplace activity limitations. Arthritis Rheum 2005;53(3):328-36.

For peer review only

41. Kievit AJ, Kuijer PPFM, Kievit RA, Sierevelt IN, Blankevoort L, Frings-Dresen MHW, A Reliable, Valid and Responsive Questionnaire to Score the Impact of Knee Complaints on Work Following Total Knee Arthroplasty: The WORQ. J ARTHROPLASTY 2014;ahead of print [PubMed]

42. Kievit AJ, van Geenen RCI, Kuijer PPFM, Pahlplatz TMJ, Blankevoort L, Schafroth MU, Total Knee Arthroplasty and the Unforeseen Impact on Return to Work: A Cross-Sectional Multicenter Survey. J ARTHROPLASTY 2014;ahead of print [PubMed]

43~~39~~. Gignac MA, Jetha A, Bowring J, Beaton DE, Badley EM. Management of work disability in rheumatic conditions: a review of non-pharmacological interventions. Best Pract Res Clin Rheumatol. 2012;26(3):369-86.

44~~40~~. Coole C, Watson PJ, Drummond A. Staying at work with back pain: patients' experiences of work-related help received from GPs and other clinicians. A qualitative study. BMC Musculoskelet Disord 2010;11:190.

Table 1: Outcome and independent variables included in the analysis

	Time point when data was collected		
	Baseline	3-year follow-up	6- year follow-up
**Outcome**			
Expected work limitation			√
**Independent factors**			
**Health factors**			
Physical function		√	
Extent of pain	√	√	√
Comorbidity	√	√	√
Smoking	√		
Depression	√	√	√
Anxiety	√	√	√
Body mass Index	√	√	√
Cognitive impairment	√	√	√
Control of health	√		
**Socio-demographic**			
Age	√		
Gender	√		
Educational attainment	√		
Occupational classification	√		
Adequacy of income	√		
Live alone	√		

For peer review only - http://bmjopen.bmj.com/site/about/guidelines.xhtml

**Workplace factors**	
Work type	√
Work status	√
Physically demands of job	√
Flexible working	√
Use of aids and appliances	√
Opportunities to retrain	√
Work satisfaction	√

Table ~~1~~2. Subject characteristics overall and by prediction status (n=297)

	All (n=297)	Will work to 69 without limitation (n=51)	Don't know if joint pain will limit (n =167)	Joint pain will limited or stop me working until 69 (n=79)
Age				
Median (Standard deviation~~error~~)	54 (2.34~~0.25~~)	57 (2.47~~0.35~~)	54 (2.35~~0.18~~)	53 (~~0.22~~2.00)
Gender				
Male	134 (45.1)	32 (60.0)	64 (38.3)	38 (48.1)
Female	163 (54.9)	19 (40.0)	103 (61.7)	41 (51.9)
Educational attainment				
Further	63 (21.5)	11 (21.6)	39 (23.8)	13 (16.7)
School only	230 (78.5)	40 (78.4)	125 (76.2)	65 (83.3)
Live alone				
No	37 (12.9)	6 (12.2)	14 (8.6)	17 (22.7)
Yes	250 (87.1)	43 (87.8)	149 (91.4)	58 (77.3)
Occupational classification				
Managerial/Professional	73 (24.~~7~~6)	17 (33.3)	40 (24.1)	16 (20.3)
Intermediate	57 (19.~~2~~3)	10 (19.6)	28 (16.9)	19 (24.1)
Routine	166 (55.9~~6.1~~)	24 (47.1)	98 (59.0)	44 (55.7)
Adequacy of income				
Adequate	129 (43.4)	22 (43.1)	85 (50.9)	22 (27.9)
Inadequate	168 (56.6)	29 (56.9)	82 (49.1)	57 (72.2)
Physical Function				
Median (Standard error)	85 (10.5)	90 (7.91)	85 (9.27)	65 (12.2)
Pain status				
None	15 (5.1)	2 (3.9)	12 (7.2)	1 (1.3)
Some	143 (48.2)	30 (58.8)	88 (52.7)	25 (31.7)
Widespread	139 (46.8)	19 (37.3)	67 (40.1)	53 (67.1)
Comorbidity				
Low comorbidity (0-3)	122 (41.1)	20 (39.2)	78 (46.7)	24 (30.4)
Multimorbidity (4 or more)	175 (58.9)	31 (60.8)	89 (53.3)	55 (69.6)
Depression				
Non-case (0-7)	228 (76.8)	44 (86.3)	138 (82.6)	46 (58.2)
Possible/probable case (8-21)	69 (23.2)	7 (13.7)	29 (17.4)	33 (41)
Anxiety				
Non-case (0-7)	121 (40.7)	27 (52.9)	65 (38.9)	29 (36.7)
Possible/probable case (8-21)	176 (59.3)	24 (47.1)	102 (61.1)	50 (63.3)
Body mass Index				
Normal (20-24.9 kg m^2)	68 (24.7)	13 (27.7)	42 (26.4)	13 (18.8)
Underweight (<20)	9 (3.3)	1 (2.1)	6 (3.8)	2 (2.9)
Overweight (25-29.9 kg m^2)	102 (37.1)	21 (44.7)	52 (32.7)	29 (42.0)
Obese (>30 kg m^2)	96 (34.9)	12 (25.5)	59 (37.1)	25 (36.2)
Cognitive complaint				
No cognitive complaint	122 (41.~~8~~1)	28 (54.9)	75 (44.9)	19 (24.1)
Cognitive complaint	175 (58.9)	23 (45.1)	92 (55.1)	60 (76.0)
Smoking				
Never	119 (40.2)	22 (44.0)	73 (43.7)	24 (30.4)
Previous	119 (40.2)	20 (40.0)	68 (40.7)	31 (39.2)
Current	58 (19.6)	8 (16.0)	26 (15.6)	24 (30.4)
Control*				
Can control health	277 (93.9)	48 (94.1)	157 (95.2)	72 (91.1)

For peer review only

For peer review only - http://bmjopen.bmj.com/site/about/guidelines.xhtml

Can't control health	18 (6.1)	3 (5.9)	8 (4.9)	7 (8.9)
Work satisfaction				
Satisfied	198 (74.2)	43 (86.0)	115 (75.2)	40 (62.5)
Dissatisfied	69 (25.8)	7 (14.0)	38 (24.8)	24 (37.5)
Work type*				
Sedentary	88 (34.1)	18 (36.7)	53 (36.1)	17 (27.4)
Standing	71 (27.5)	9 (23.1)	41 (27.9)	21 (33.9)
Physical	81 (31.4)	20 (38.9)	45 (30.6)	16 (25.8)
Heavy manual	18 (7.0)	2 (4.6)	8 (5.4)	8 (12.9)
Physically demands of job				
Not physically demanding	171 (57.6)	36 (70.6)	101 (60.5)	34 (43.0)
Physically demanding	126 (42.4)	15 (29.4)	66 (39.5)	45 (57.0)
Flexible working				
Flexible	82 (27.6)	19 (37.3)	39 (23.4)	24 (30.4)
Not flexible	215 (72.4)	32 (62.78)	128 (76.67)	55 (69.6)
Use of aids and appliances				
Yes	151 (50.8)	32 (62.78)	79 (47.3)	40 (50.6)
No	146 (49.2)	19 (3.7.3)	88 (52.7)	39 (49.4)
Opportunities to retrain				
Yes	205 (69.0)	42 (82.4)	115 (68.9)	48 (60.8)
No	92 (31.0)	9 (17.67)	52(31.1)	31 (39.2)
Co-worker support				
Good co-worker support	277 (93.3)	51 (100)	154 (92.2)	72 (91.1)
Low co-worker support	20 (6.7)	0 (0)	13 (7.8)	7 (8.9)
Work amount				
Full-time	138 (46.5)	31 (60.8)	70 (41.9)	37 (46.8)
Part-time	121 (40.7)	15 (29.4)	81 (48.5)	25 (31.7)
Temp. work absence	38 (12.8)	5 (9.8)	16 (9.6)	15 15 (21.5)

*Missing data

Table 32. Associations between health, demographic, socio-economic and workplace factors and significant future work limitation in primary care consulters for osteoarthritis; comparing EWL with no EWL odds ratios with 95% confidence intervals

			Multivariate model within each domain		Multivariate model including all domains	
	OR*	95%CI†	OR	95%CI	OR	95%CI
Health factors						
Physical function	**0.93**	**0.91, 0.96**	**0.94**	**0.91, 0.97**	**0.95**	**0.92, 0.97**
Extent of pain						
No pain	1		1		-	
Some	1.67	0.14, 19.5	0.44	0.03, 6.76	-	
Widespread	5.58	0.48, 65.1	0.84	0.05, 13.74	-	
Comorbidity						
Low comorbidity (0-3)	1		1		-	
Multimorbidity (4 or more)	1.48	0.71, 3.10	0.97	0.37, 2.56	-	
Smoking						
Never	1		1		1	
Previously	1.42	0.63, 3.18	2.19	0.73, 6.53,	1.52	0.51, 4.51
Currently	**2.75**	**1.02, 7.38**	2.99	0.79, 11.30	2.02	0.55, 7.40
Depression						
Non-case (0-7)	1		1		-	
Possible/probable case (8-21)	**4.51**	**1.81, 11.3**	1.27	0.35, 4.63	-	
Anxiety						
Non-case (0-7)	1		1		-	
Possible/probable case (8-21)	1.94	0.95, 3.97	1.08	0.37, 3.19	-	
Body mass Index						
Normal (20-24.9 kg m^2)	1		1		-	
Underweight (<20)	1.80	0.14, 23.4	1.15	0.02, 64.92	-	
Overweight (25-29.9 kg m^2)	1.13	0.39, 3.21	1.29	0.33, 5.09	-	
Obese (>30 kg m^2)	2.08	0.71, 6.10	1.33	0.31, 5.66	-	
Cognitive impairment						
No cognitive complaint	1		1		-	

Formatted: Numbered + Level: 1 + Numbering Style: 1, 2, 3, … + Start at: 15 + Alignment: Left + Aligned at: 0.25" + Indent at: 0.5"

Formatted: Indent: Left: 0.5"

Formatted: Font: Not Bold

Formatted: Font: Bold

Formatted: Font: Bold

Formatted: Font: Bold

Formatted: Font: Bold

Formatted: Font: Bold

Formatted: Font: Bold

Formatted: Font: Bold

For peer review only - http://bmjopen.bmj.com/site/about/guidelines.xhtml

Cognitive complaint	**3.84**	**1.81, 8.18**	1.98	0.70, 5.58	-	
Control of health						
Can control health	1		1		-	
Can't control health	1.56	0.38, 6.31	1.64	0.29, 9.36	-	
R^2			0.311			
C- statistic			0.8641			
Socio-demographic						
Age	0.69	0.58, 0.82	0.67	0.56, 0.81	**0.74**	**0.60, 0.91**
Gender						
Male	1		1		-	
Female	1.82	0.89, 3.72	1.37	0.58, 3.22	-	
Educational attainment						
Further	1		1		-	
School only	1.38	0.56, 3.36	1.12	0.34, 3.64	-	
Occupational classification						
Managerial/Professional	1		1		-	
Intermediate	2.01	0.72, 5.63	2.27	0.64, 8.12	-	
Routine	1.94	0.84, 4.53	2.26	0.74, 6.90	-	
Adequacy of income						
Adequate	1		1		-	
Inadequate	1.97	0.94, 4.12	2.01	0.84, 4.81	-	
Live alone						
No	1		1		-	
Yes	0.48	0.17, 1.31	0.50	0.16, 1.56	-	
R^2			0.175			
C-statistic			0.7636			
Workplace factors						
Work type						
Sedentary	1		1		-	
Standing	2.47	0.89, 6.88	2.32	0.60, 7.92	-	
Physical	0.85	0.33, 2.15	0.46	0.13, 1.59	-	
Heavy manual	4.24	0.79, 22.8	2.38	0.30, 18.7	-	
Work status						
Full-time	1		1		-	
Part-time	1.40	0.63, 3.10	1.07	0.40, 2.87	-	
Off work	2.85	0.94, 8.60	0.26	0.04, 1.91	-	
Physically demands of job						
Not physically demanding	1		1		-	
Physically demanding	3.18	1.50, 6.72	1.81	0.63, 5.21	-	
Flexible working						
Flexible	1		1		-	
Not flexible	1.36	0.65, 2.86	1.77	0.62, 5.04	-	
Use of aids and appliances						
Yes	1		1		-	
No	1.64	0.80, 3.37	1.06	0.43, 2.65	-	
Opportunities to retrain						
Yes	1		1		-	
No	3.01	1.29, 7.05	1.99	0.72, 5.45	-	
Work satisfaction						
Satisfied	1		1		1	
Dissatisfied	3.69	1.43, 9.49	4.78	1.57, 14.6	2.08	0.66, 6.51
R^2			0.116		0.296	
C-statistic			0.7785		0.8418	

*Odds ratio; †95 percent confidence interval;

Formatted: Font: Bold

Formatted: Superscript

Formatted: Font: Bold

Formatted: Font: Bold

Formatted: Superscript

Formatted: Superscript

For peer review only - http://bmjopen.bmj.com/site/about/guidelines.xhtml

Figure 1. Flow diagram of participants for the longitudinal analysis

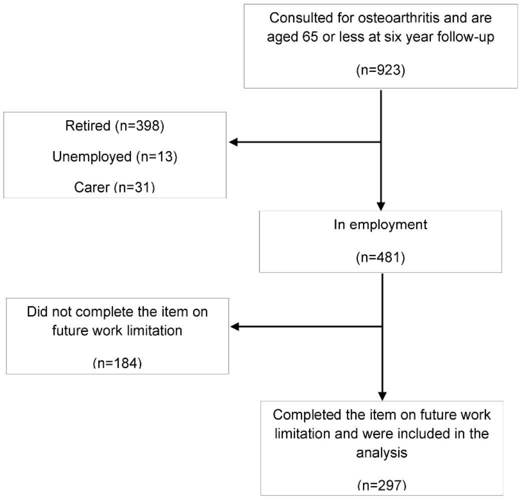

1

297x420mm (300 x 300 DPI)

STROBE Statement—Checklist of items that should be included in reports of ***cohort studies***

	Item No	Recommendation
**Title and abstract**	1	(*a*) Indicate the study's design with a commonly used term in the title or the abstract<br><br>The title of the study is: "Anticipated significant work limitation in primary care consulters with osteoarthritis: a prospective cohort study"
		(*b*) Provide in the abstract an informative and balanced summary of what was done and what was found<br><br>The abstract summarises the methods and key results
**Introduction**		
Background/rationale	2	Explain the scientific background and rationale for the investigation being reported<br><br>The scientific background and rationale for the investigation are reported in the introduction (see page 3).<br>"Osteoarthritis is the most common joint condition in adults and globally is the fastest increasing major heath condition [1]. It is a common reason for primary care consultation (one in twenty consultations in adults aged between 45 and 65 per year is for osteoarthritis [2]) and is recognised as one of the leading and rapidly growing causes of disability [3]. Its most disabling manifestation (joint pain) is strongly associated with ageing [4] and with the commonest forms of disability [5-9].<br>Work restriction is one form of disability that will become more important for those with osteoarthritis and joint pain because increases in state pension age in many developed countries means that most adults can expect a need to continue working at older ages than before [10]. Normal retirement age in North America and Europe has increased, and is expected to rise further to 69 and beyond [11]. However the extent to which participation in work will be limited by health-related problems, resulting in significant work limitation in terms of absenteeism and presenteeism (remaining in work but with limitation and reduced productivity) is unclear [12]. The increasing prevalence of chronic health conditions, especially osteoarthritis, in persons near to retirement age raises questions about viability of attempts to extend working life. Several studies of expectations of future work loss are predictive of future work outcomes [13,14]. Identifying the prevalence and predictors of expected work limitations (EWL) in this group of patients, particularly those that are amenable to change, will inform management and possible preventative strategies for future work limitation."
Objectives	3	State specific objectives, including any prespecified hypotheses<br><br>Objectives of the study are stated in the last paragraph of the Introduction on page 3-4.

For peer review only

For peer review only - http://bmjopen.bmj.com/site/about/guidelines.xhtml

		The aim of this study was to estimate the proportion of working age adults with osteoarthritis who predict that joint pain would limit their work or stop them working (i.e. EWL) prior to a possible future pension age of 69. In addition health, socio-demographic or workplace factors associated with EWL, especially those amenable to change, were explored, to identify potential targets to manage and prevent EWL [15-17].
**Methods**		
Study design	4	Present key elements of study design early in the paper<br><br>Key elements of study design are presented in the first sentence of the Study Design paragraph in the Method section.<br>"The North Staffordshire Osteoarthritis project (NorStOP) is a population-based prospective cohort study."
Setting	5	Describe the setting, locations, and relevant dates, including periods of recruitment, exposure, follow-up, and data collection<br><br>Please see the Study Design paragraph in the Method section, as quoted in 4.
Participants	6	(*a*) Give the eligibility criteria, and the sources and methods of selection of participants. Describe methods of follow-up<br><br>Same as above. Please also see Figure 1 and the first paragraph of the Results section
		(*b*) For matched studies, give matching criteria and number of exposed and unexposed<br><br>Not applicable
Variables	7	Clearly define all outcomes, exposures, predictors, potential confounders, and effect modifiers. Give diagnostic criteria, if applicable<br><br>Please see the sections spanning from page 5 to page 8 (Identification of osteoarthritis to the end of workplace factors).
Data sources/ measurement	8*	For each variable of interest, give sources of data and details of methods of assessment (measurement). Describe comparability of assessment methods if there is more than one group<br><br>Same as 7.
Bias	9	Describe any efforts to address potential sources of bias<br><br>Additional analysis was undertaken to examine the potential impact of missing data (non-completion of the outcome question) (see paragraph 2 of the methods).

For peer review only

Study size	10	Explain how the study size was arrived at<br>The derivation of the study sample is explained in the second paragraph of the methods section<br>The study sample was based on those who (i) consulted for osteoarthritis during the study period (starting 18 months before the baseline questionnaire was administered, and continuing through the time of the final follow-up questionnaire (i.e. from 2000 to 2008)), (ii) were of working age (less than 65 years old) and in employment at the six year follow-up and (iii) completed the item on expected work limitation prior to 69 years old (EWL) at six year follow-up.
Quantitative variables	11	Explain how quantitative variables were handled in the analyses. If applicable, describe which groupings were chosen and why<br>This is outlined in the Statistical Analysis section on pages 8.
Statistical methods	12	(*a*) Describe all statistical methods, including those used to control for confounding<br>This is outlined in the Statistical Analysis section on pages 8
		(*b*) Describe any methods used to examine subgroups and interactions<br>Not applicable
		(*c*) Explain how missing data were addressed<br>Patients were included only if they had complete data
		(*d*) If applicable, explain how loss to follow-up was addressed<br>The potential bias due to loss of participants is summarised in the paper
		(*e*) Describe any sensitivity analyses<br>Same as 12(d)
**Results**		
Participants	13*	(a) Report numbers of individuals at each stage of study—eg numbers potentially eligible, examined for eligibility, confirmed eligible, included in the study, completing follow-up, and analysed<br>These are described in the text and if further illustrated in figure 1.
		(b) Give reasons for non-participation at each stage<br>The reasons for non-participation are provided at each stage
		(c) Consider use of a flow diagram

For peer review only - http://bmjopen.bmj.com/site/about/guidelines.xhtml

		See Figure 1
Descriptive data	14*	(a) Give characteristics of study participants (eg demographic, clinical, social) and information on exposures and potential confounders<br><br>See the first paragraph of the results and Table 1
		(b) Indicate number of participants with missing data for each variable of interest<br><br>The analysis has been done in those with complete data
		(c) Summarise follow-up time (eg, average and total amount)<br><br>Data included in this study has been collected over 6 years. Please see methods section.
Outcome data	15*	Report numbers of outcome events or summary measures over time<br><br>Results in the text and tables adhere to these guidelines.
Main results	16	(*a*) Give unadjusted estimates and, if applicable, confounder-adjusted estimates and their precision (eg, 95% confidence interval). Make clear which confounders were adjusted for and why they were included<br><br>Results in the text and tables adhere to these guidelines. See also Table 2
		(*b*) Report category boundaries when continuous variables were categorized<br><br>Same as 16(a)
		(*c*) If relevant, consider translating estimates of relative risk into absolute risk for a meaningful time period<br><br>Not applicable.
Other analyses	17	Report other analyses done—eg analyses of subgroups and interactions, and sensitivity analyses<br><br>All analyses are fully reported in the statistical methods section
**Discussion**		
Key results	18	Summarise key results with reference to study objectives<br><br>This is done in the first paragraph (pages 11-12)
Limitations	19	Discuss limitations of the study, taking into account sources of potential bias or imprecision. Discuss both direction and magnitude of any potential bias

For peer review only

		This is done in the strengths and limitations section of the discussion (pages 12-13)
Interpretation	20	Give a cautious overall interpretation of results considering objectives, limitations, multiplicity of analyses, results from similar studies, and other relevant evidence<br><br>This is done in the comparison with existing literature section of the discussion (pages 10-15)
Generalisability	21	Discuss the generalisability (external validity) of the study results<br><br>This is done in the strengths and limitations section of the discussion (pages 12-13)
**Other information**		
Funding	22	Give the source of funding and the role of the funders for the present study and, if applicable, for the original study on which the present article is based<br><br>This information is disclosed at the end of the manuscript

*Give information separately for exposed and unexposed groups.

Note: An Explanation and Elaboration article discusses each checklist item and gives methodological background and published examples of transparent reporting. The STROBE checklist is best used in conjunction with this article (freely available on the Web sites of PLoS Medicine at http://www.plosmedicine.org/, Annals of Internal Medicine at http://www.annals.org/, and Epidemiology at http://www.epidem.com/). Information on the STROBE Initiative is available at http://www.strobe-statement.org.

For peer review only

For peer review only - http://bmjopen.bmj.com/site/about/guidelines.xhtml
